# Blockade of TGF-β and PD-L1 by bintrafusp alfa promotes survival in preclinical ovarian cancer models by promoting T effector and NK cell responses

**DOI:** 10.1038/s41416-024-02677-9

**Published:** 2024-04-15

**Authors:** Jacob Kment, Daniel Newsted, Stephanie Young, Michael C. Vermeulen, Brian J. Laight, Peter A. Greer, Yan Lan, Andrew W. Craig

**Affiliations:** 1 Cancer Biology & Genetics division, Queen’s Cancer Research Institute, Kingston, ON Canada; 2 Department of Biomedical and Molecular Sciences, Queen’s University, Kingston, ON Canada; 3 Department of Pathology and Molecular Medicine, Queen’s University, Kingston, ON Canada; 4 EMD Serono Research & Development Institute, Inc., Billerica, MA USA

**Keywords:** Ovarian cancer, Cancer immunotherapy

## Abstract

**Background:**

Failure of immunotherapy in high-grade serous ovarian cancer (HGSC) may be due to high levels of transforming growth factor-β (TGF-β) in ascites or tumour immune microenvironment (TIME). Here, we test whether coordinated blockade of TGF-β and PD-L1 with bintrafusp alfa (BA) can provoke anti-tumour immune responses in preclinical HGSC models.

**Methods:**

BA is a first-in-class bifunctional inhibitor of TGF-β and PD-L1, and was tested for effects on overall survival and altered TIME in syngeneic HGSC models.

**Results:**

Using a mouse ID8-derived HGSC syngeneic model with IFNγ-inducible PD-L1 expression, BA treatments significantly reduced ascites development and tumour burden. BA treatments depleted TGF-β and VEGF in ascites, and skewed the TIME towards cytotoxicity compared to control. In the BR5 HGSC syngeneic model, BA treatments increased tumour-infiltrating CD8 T cells with effector memory and cytotoxic markers, as well as cytolytic NK cells. Extended BA treatments in the BR5 model produced ∼50% BA-cured mice that were protected from re-challenge. These BA-cured mice had increased peritoneal T-effector memory and NK cells compared to controls.

**Conclusions:**

Our preclinical studies of BA in advanced ovarian cancer models support further testing of BA as an improved immunotherapy option for patients with advanced ovarian cancer.

## Introduction

Ovarian cancer is the most lethal gynaecological malignancy, and high-grade serous carcinoma (HGSC) is the most prevalent subtype [1]. Patients with HGSC are often diagnosed with advanced metastatic disease that is associated with higher mortality rates compared to localised early-stage disease [1–3]. Patients undergo debulking surgery followed by platinum-based chemotherapy, and although initial responses exceed 75%, most patients experience recurrence of platinum-resistant tumours and progression-free survival of only 12–18 months [4, 5]. Developing improved systemic therapies such as immunotherapies for recurrent HGSC will be critical to improve patient survival.

Immunotherapies that modulate host immune responses to ovarian tumours have been tested. Of these, immune checkpoint inhibitors (ICI) that target PD-L1/PD-1 and CTLA-4/CD80 immune suppressive pathways have been studied [6]. Although these ICI have become standard of care in advanced NSCLC [7], melanoma [8, 9], and urothelial cancers [10, 11], the results from early clinical trials in ovarian cancer revealed very low response rates as monotherapies (10–25%) [12]. However, in patients that experienced a treatment response, profound disease control was observed [13, 14]. To overcome limitations with ICI monotherapies, clinical trials examining double checkpoint blockades and ICI combinations with chemotherapy, PARP inhibitors, bevacizumab or tyrosine kinase inhibitors have been initiated [6]. Studies that identify potential barriers to immunotherapy responses in HGSC are needed to inform on optimal treatments for HGSC patients.

Transforming growth factor beta (TGF-β) is a pleiotropic cytokine that may contribute to HGSC progression and resistance to current therapies. Preclinical studies have revealed that TGF-β drives tumour cell migration and metastatic invasion [15, 16], cancer stem cell maintenance [17], chemoresistance [18, 19] and immunosuppression [20–23]. Pretreatments of mice with patient-derived xenograft tumours with TGFBR1 inhibitor galunisertib/LY2157299 led to reduced tumour growth and ascites development [24], suggesting potential as a maintenance therapy to limit recurrence. Previously, we developed synthetic TGFBR2 antibodies by phage display screening and showed that these inhibitory antibodies could limit tumour growth and immune exclusion in immunocompetent HGSC mouse models [25]. In humans, TGF-β expression in ovarian tumours is associated with immunosuppression, poor prognosis and reduced survival [26–28]. However, there are currently no clinically approved treatments targeting TGF-β as monotherapy, or in combination with other therapies for HGSC.

Bintrafusp alfa (BA; previously called M7824) is a first-in-class bifunctional fusion protein composed of the extracellular domain of the human TGF-β receptor II (TGF-β “trap”) fused via a flexible linker to the C-terminus of each heavy chain of an IgG_1_ antibody inhibitor of PD-L1. In preclinical breast cancer and colon cancer models, BA treatment dramatically reduced tumour growth due to increased activation of T and NK cells, leading to the development of immunological memory [29, 30]. Combined with 5-fluorouracil or radiotherapy, BA produces an even more substantial reduction in tumour burden than either modality alone [30]. In NSCLC xenograft models, BA was effective in diminishing mesenchymal signatures and limiting tumour growth [31]. In a recent Phase I clinical trial, BA-treated patients had manageable adverse effects, and no maximum tolerable dose was identified through dose escalation [32]. Despite its recent success in several tumour types [33–35], the impact of BA has not been examined in ovarian cancer.

In this study, the efficacy of BA was tested for the first time in mouse syngeneic models of metastatic HGSC. Using mouse ID8-Trp53^−/−^:Brca2^−/−^ double knock-out (ID8-DKO) as a syngeneic HGSC model [36], BA treatments over 6 days failed to provide significant survival benefit. However, with prolonged BA treatments in B-cell-deficient mice engrafted with this model, we observed a significant survival benefit, reduced tumour burden and ascites compared to control treatments. Tumour profiling revealed that BA treatments increased M1 tumour-associated macrophages (TAMs), and gene signatures associated with cytotoxicity and immune activation. BA treatments were even more effective in the BR5/FVB syngeneic HGSC model, with evidence of tumour regressions, infiltration by cytotoxic T and NK cells, and 50% achieving long-term survival (BA-cured). These BA-cured mice also rejected BR5 tumours upon re-challenge without further treatments. This correlated with expanded T-effector memory and NK cells in BA-cured mice compared to naive mice.

## Materials and methods

### Cell culture and treatments

Mouse HGSC ID8-Trp53^−/−^:Brca2^−/−^ double knock-out (ID8-DKO) cells [36] were transduced with a lentiviral pWPI-GFP/Luciferase dual reporter (ID8-DKO-Luc). We also acquired mouse HGSC BR5-Trp53^−/−^:Brca1^−/−^:Tg-Myc:Tg-Akt cells transduced with a Luciferase reporter (BR5-Luc; ref. [37]). Both cell lines were cultured in DMEM supplemented with 10% FB Essence (VWR) and penicillin–streptomycin (Sigma-Aldrich). To examine PD-L1 surface expression on both cell lines, we stimulated the cells with either TGF-β1 (5 ng/mL, Peprotech), IFNγ (20 ng/mL, Peprotech) or a combination of both for 72 h. Cells were lifted from wells by treatment with 5% EDTA, washed with PBS, Fcγ receptors were blocked with anti-CD16/32 antibody (Biolegend) for 30 min prior to incubation with an anti-PD-L1-PE-Cy7 (Biolegend 124313, clone 10 F.9G2) antibody for 1 h at 4 °C. Flow cytometry was performed in triplicate with a Cytoflex Flow Cytometer (BeckmanCoulter) using V-bottom 96-well plates (Sartedt).

### Syngeneic HGSC mouse models

Two syngeneic engraftment models were used to mimic HGSC metastasis in the peritoneal cavity. First, ID8-DKO-Luc cells were passaged in growth media while at ≤80% confluency, and live cells were counted using a hemocytometer. For tumour engraftment studies, 5 million cells were delivered by intraperitoneal (i.p.) injection in female 6–8 week-old C57BL/6 mice (Charles River), or B-cell-deficient µMT^−^ mice (B6.129S2-Ighm homozygous, catalogue number 002288, Jackson Labs). BR5-Luc cells were passaged similar to ID8-DKO-Luc cells. For in vivo work, four million BR5-Luc cells were injected i.p. in female 6–8 week-old FVB mice (Charles River). In experiments with >3 treatments, B cells were depleted with anti-CD20 i.p. injections (250 µg, BioXCell BE0356) in FVB mice every 3 weeks. For both syngeneic models, biophotonic imaging (IVIS) was performed weekly by injecting d-Luciferin (3 mg in 0.2 ml PBS i.p.) followed by anaesthetising mice with isoflurane, and imaging on a Pearl Trilogy Small Animal Imaging System (LICOR). Quantification of IVIS was performed using Living Image Software (PerkinElmer). For the ID8-DKO model, mice were randomised into eight mice per treatment group on day 15. Mice were initially treated three times with control IgG (400 µg) or BA (492 µg) at the beginning of week 3 (days 21, 23, 25) in a pilot study that compared the survival of immunocompetent C57BL/6 mice. In an extended follow-up survival study in µMT^-^ mice, control IgG (400 µg) or BA (492 µg) treatments were given i.p. twice weekly for 4 weeks and allowed to reach protocol-defined endpoints. In the endpoint study, control IgG (400 µg), TGF-β Trap (inactive anti-PD-L1 fused to the extracellular domain of TGF-βRII; 492 µg), anti-PD-L1 (400 µg) and BA (492 µg) were given by i.p. injections twice weekly for 4 weeks (note that these are equimolar doses of these proteins). At day 45, mice were sacrificed, ascites fluid and metastatic tumour nodules were collected. For mice that had low ascites volumes, peritoneal lavage was performed with a known volume of saline and a dilution factor for that ascites sample was calculated for subsequent analyses.

For engraftment studies with the BR5-Luc model, a pilot survival study was conducted using female FVB mice (10/group) injected with BR5-Luc cells (day 0) followed by treatments with control IgG (400 µg) or BA (492 µg) on days 11, 13, and 15. Mice were removed upon reaching protocol-defined endpoints. For an extended treatment regime and survival study, female FVB mice injected with BR5-Luc cells were treated twice weekly with control IgG (400 μg) or BA (492 μg) i.p. with B-cell depletion using anti-CD20 (described above). Mice were sacrificed when they met protocol-defined endpoints. Surviving mice from BA group (BA-cured), along with age-matched treatment-naive FVB mice (Charles River), were re-challenged with 4 million BR5-Luc cells i.p. without additional treatments or anti-CD20 injections. At day 107, a peritoneal lavage was performed to profile immune cells. Flow cytometry was performed to compare effector memory T cells and NK cell populations between treatment groups. In the BR5/FVB endpoint study, mice were injected with 4 million BR5-Luc cells i.p. on day 0 without B-cell depletion. Tumour burden was monitored weekly with IVIS over 7 weeks to ensure that all mice had similar tumour burden. At day 49, mice were randomised into 6 mice per treatment group. Treatments of control IgG (400 µg), TGF-β Trap (492 µg), anti-PD-L1 (400 µg) and BA (492 µg) were administered on days 52, 54, and 56. On the morning of day 59, mice were sacrificed, and peritoneal lavage/ascites and metastatic tumour nodules were collected for further analyses. All animal studies were performed in accordance with protocols approved by the Queen’s University Animal Care Committee that reflect guidelines from the Canadian Council on Animal Care.

### Ascites cytokine analysis

Ascites were collected from peritoneum either directly, or after lavage with a known volume of saline. Ascite cells were removed by centrifugation, and ascite fluid was aliquoted and frozen on dry ice. To measure cytokine levels in the ascites fluid, samples were diluted in an equal volume of saline, and analysed using a TGF-β 3-Plex (TGFB1-3) panel and a mouse cytokine 31-Plex panel (MD31, Eve Technologies). The dilution of samples was corrected for prior to the analysis of cytokine concentration data using GraphPad Prism.

### pSmad immunoblotting

For cell line experiments, 1 × 10^6^ ID8-DKO or BR5 cells were cultured in six-well plates and exposed to 5 ng/mL TGF-β1 (Peprotech) with or without LY2157299 (SelleckChem) for 1 h prior to cell lysis. For analysis of BA treatments in vivo, ascites cells or 0.1 mg of tumour tissue was homogenised in lysis buffer. In all experiments, lysis buffer consisted of RIPA Buffer (25 mM Tris pH 7.5, 150 mM NaCl, 1% NP-40, 1% SDS, 0.5% sodium deoxycholate) supplemented with phosphatase and protease inhibitors (1 mM Na_3_VO_4_, 100 μM phenylmethylsulfonyl fluoride, 10 μg/mL aprotinin, 10 μg/mL leupeptin and 50 mM NaF). To examine the extent of Smad activation, anti-phospho-Smad2/3 (pSmad, clone D27F4, CST, 1:1000) and pan-Smad2/3 (clone D7G7, CST, 1:1000) primary antibodies (along with anti-α-Actinin loading control) were used in conjunction with an anti-rabbit-HRP secondary antibody (CST, 1:25,000). ECL Western Blotting Substrate (Thermo Scientific) was used to detect protein levels. ImageJ software was used for densitometry analysis of the pSmad2/3:Smad2/3 ratio.

### Immunophenotyping and immunostaining

To characterise activation states of ascites T cells and tumour cells, ascites samples were subjected to red cell lysis (Biolegend) prior to counting. One million cells were treated with anti-CD16/32 (Invitrogen 14-0161-82) and 100,000 cells were distributed in several wells of a multiwell plate. For the ID8-DKO-Luc endpoint, primary antibodies targeting surface antigens such as CD45-PE/Cy7 (BD 561868, clone 30-F11,), CD8b-PE (Biolegend 126607, clone YTS156.7.7), CD4-PE/Cy5 (Biolegend 100513, clone RM4-5), CD25-PE (Biolegend 101903, clone 3C7), CD152/CTLA-4-APC (Biolegend 106309, clone UC10-4B9), CD326/EpCAM-PE (Biolegend 118205, clone G8-8), PD-L1-APC (Biolegend 124311, clone 10F.9G2), LY6G-PE (Biolegend 127607, clone 1A8), LY6C-FITC (BD 553104, clone AL-21) and CD11b-PE/Cy5 (Biolegend 101209, clone M1/70) were used as well as intracellular staining for FOXP3-AF488 (Biolegend 126405, clone MF-14). Additions to the aforementioned antibody panels that were used for the BR5-Luc peritoneal lavage include primary antibodies targeting CD45-FITC (Biolegend 103107, clone 30-F11), CD44-AF700 (Biolegend 103025, clone IM7), CD62L-BV605 (Biolegend 104437, clone MEL-14) and CD49b-PE/Cy7 (Biolegend 108921, clone DX5). These antibody panels were expanded further to incorporate staining of: CD4-APC (Biolegend 116013, clone RM4-4), LAMP-1/CD107a-PE (Biolegend 121611, clone 1D4B), CD27-BV605 (Biolegend 124249, clone LG.3A10), CD11b-APC (Biolegend 101211, M1/70), CD279/PD-1-PE/Cy7 (Biolegend 135215, clone 29F.1A12), Chrome-orange LIVE/DEAD Fixable Aqua Dead Cell Stain Kit (Invitrogen L34966), and intracellular staining with IFN-γ-PE/Cy7 (Biolegend 505825, clone XMG1.2) or FOXP3-PE (Biolegend 126403, clone MF-14). Intracellular staining was performed according to Foxp3/Transcription Factor Staining Buffer Set (eBioscience, 00-5523-00). Prior to staining, tumours from the BR5/FVB model were mechanically dissociated and digested using Collagenase B (1 mg/mL) and DNase I (25 μg/mL) and then transferred to *Miltenyi Biotec* C-tubes. Samples were processed using the TDK1 programme on the gentleMACS (*Miltenyi Biotec*) dissociator for 41 min and then passed through both 70- and 40-µm cell strainers followed by red blood cell lysis and counting prior to antibody staining and flow cytometry (see Supplementary Fig. S8 for gating strategies). The extent of B-cell depletion was assessed using flow cytometry with primary antibodies targeting CD45R/B220-FITC (Biolegend 103205, clone RA3-6B2) and CD19-PE (Biolegend 152407, clone 1D3/CD19). All flow cytometry was performed with a Cytoflex Flow Cytometer (BeckmanCoulter) in V-bottom 96-well plates (Sarstedt). Tumour nodules were prepared for cryosectioning, and 20-μm thick sections were post-fixed in acetone and blocked for 1 h with 3% bovine serum albumin prior to the addition of primary antibodies overnight at 4 °C. Sources and dilutions of primary antibodies were as follows: AF488 rat anti-mouse CD68 (Biolegend 137011, clone FA-11; 1:100), AF594 rat anti-mouse CD206 (Biolegend 141726, clone C068C2; 1:100) and AF594 rat anti-mouse iNOS (Biolegend 696803, clone W16030C; 1:300). DAPI (1:400) was also included to detect cell nuclei, and images were acquired by epifluorescence microscopy (EVOS M7000, Invitrogen). Analysis of CD68^+^, CD206^+^ and iNOS^+^ area relative to DAPI was performed using ImageJ and analysed in GraphPad Prism.

### RNA extraction and nanostring profiling

To compare gene expression across treatment groups, solid tumour nodules collected from each mouse were ground in liquid nitrogen using a mortar and pestle. RNA was extracted from 100 µg of ground tumour tissue using Nordic RNA Extraction Kit (Nordic Biosite). Extracted RNA was quantified using a BioAnalyzer and 150 ng was hybridised to the PanCancer Immune Profiling Panel (Nanostring Technologies). Counting of fluorescent probes to quantify target RNA molecules was performed using an nCounter Digital Analyzer (Queen’s Laboratory for Molecular Pathology, Queen’s University). Data normalisation and gene expression analysis was conducted using nSolver 4 software (Nanostring). The Advanced Analysis feature within nSolver enabled mapping of differentially expressed genes to biological pathways and cell types. Differential expression data was visualised using R (v4.1.2), and volcano plots were produced using EnhancedVolcano (v1.13.2).

### Statistical analyses

Most statistical analyses comparing treatment groups used ANOVA, or two-way ANOVA, with Tukey’s multiple comparison testing (GraphPad Prism). A test for outlier data points was conducted using Grubbs’ analysis (GraphPad Prism) prior to the removal of any samples for flow cytometry results. Kaplan–Meier plots were analysed with log-rank and Gehan–Wilcoxon tests (GraphPad Prism). Results were considered statistically significant at *P* < 0.05, and denoted in figures by *P < 0.05, **P < 0.01, ***P < 0.001 or ****P < 0.0001. All in vitro studies were replicated at least three times with similar results observed. All in vivo studies were performed once with cohort sizes predicted to give 50% power to detect 40% differences between groups (alpha 0.05).

## Results

### The ID8-derived HGSC model expresses the targets of BA

**Fig. 1:**
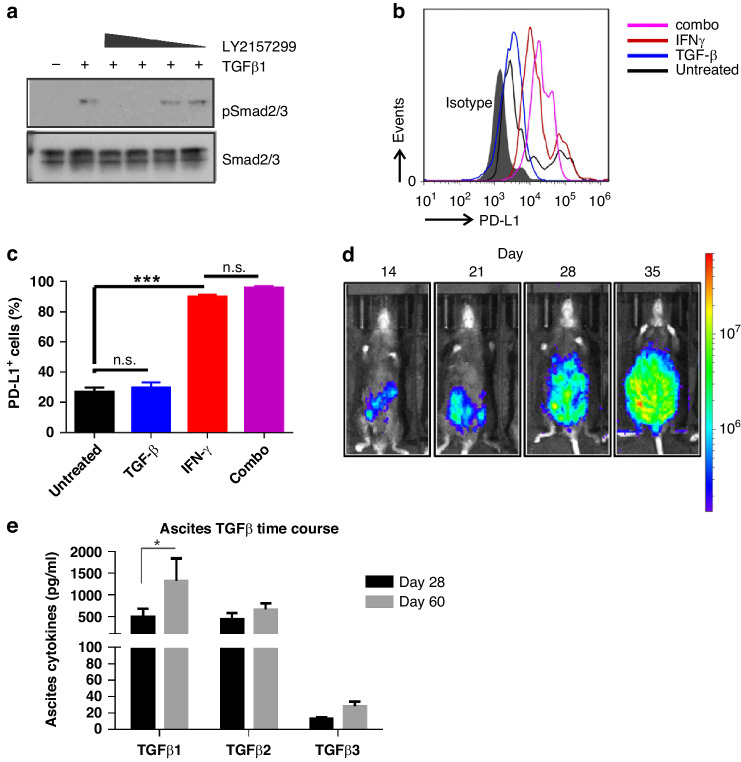
Characterisation of the syngeneic ID8-DKO mouse HGSC model for the evaluation of BA. **a** To examine the sensitivity of ID8-DKO cells to TGF-β inhibition, cells were starved overnight prior to TGF-β1 (5 ng/µL) stimulation for an hour in the absence or presence of LY2157299 (1 μM, 100 nM, 10 nM or 1 nM). Phosphorylation of Smad2/3 (pSmad2/3) compared to total Smad2/3 was analysed by immunoblotting to measure TGF-β pathway activation. **b** Flow cytometry was used to measure PD-L1 surface expression in response to TGF-β1 (5 ng/µL) or IFN-γ (20 ng/µL) exposure for 72 h in ID8-DKO cells. **c** The graph depicts a quantification of the percentage of cells expressing PD-L1 (*P < 0.05 based on unpaired *T* test). **d** ID8-DKO-Luc cells (5 × 10^6^) were injected into the peritoneum of µMT^−^ mice. Luminescence measurements were initiated at day 14 and continued weekly using an IVIS Spectrum In Vivo Imaging System. **e** Mice were sacrificed at days 28 and 60 following ID8-DKO-Luc cell injection to determine the concentration of TGF-β ligands in the ascites (*P < 0.05 based on unpaired *T* test).

To study the impact of BA on HGSC progression, we required an immune-competent murine model of HGSC that was responsive to TGF-β and expressed PD-L1. We tested mouse ID8 ovarian carcinoma cells with CRISPR/Cas9-mediated deletion of both Trp53 and Brca2 (ID8-DKO [36]). Co-treatment with TGF-β1 and increasing doses of TGFBR1 inhibitor LY2157299/galunisertib (0.1–10 µM) for an hour led to a dose-dependent suppression of Smad2/3 phosphorylation (Fig. 1a). Next, we tested the levels of surface PD-L1 expression on ID8-DKO cells that were grown in the absence or presence of TGF-β1, IFN-γ or the combination for 72 h. In untreated cells, PD-L1 levels were low and heterogeneous, but above the levels of an isotype control (Fig. 1b). In TGF-β1-treated cells, the levels of PD-L1 on the cell surface was markedly higher and less heterogeneous (Fig. 1b). Further, PD-L1 surface levels were significantly increased upon IFN-γ stimulation, and these levels were maintained in the presence of both IFN-γ and TGF-β (Fig. 1c). Since ID8-DKO cells met our requirements of being TGF-β responsive and PD-L1 expressing in vitro, we created a derivative cell line expressing Luciferase following lentiviral transduction (ID8-DKO-Luc cells). Peritoneal metastatic HGSC was modelled by syngeneic engraftment of ID8-DKO-Luc cells injected intraperitoneally (i.p.) in C57BL/6 mice, and weekly tracking was performed using biophotonic (IVIS) imaging of D-Luciferin-injected mice. These representative images depict the progression from signal in one or more nodules at early times, to a more uniform high level of radiance throughout the abdomen of the C57BL/6 mice by day 35 (Fig. 1d). To measure the levels of TGF-β ligands in the ascites fluid of this model, we harvested ascites fluid on day 28 and 60. The levels of TGF-β1 in the ascites was highest amongst family members, and significantly increased over the course of 60 days (Fig. 1e). Overall, these results identify ID8-DKO-Luc cells as an excellent model to study BA treatments since HGSC tumours are exposed to high levels of TGF-β in ascites during HGSC progression.

### Extended treatments with BA limited HGSC tumour burden and improved survival times

To begin testing of BA treatments in HGSC, we performed a survival study in female C57BL/6 mice injected with ID8-DKO-Luc cells (i.p.) prior to 3 treatments with either control IgG or BA (days 21, 23, and 25; Supplementary Fig. S1a). The BA dosage, and duration of treatment in immune-competent mice, was based on a prior study to limit antibody responses that may neutralise BA [30]. Weekly IVIS imaging, as a surrogate for HGSC tumour burden, revealed no significant differences in IVIS signal between treatment groups (Supplementary Fig. S1b). Longitudinal tracking of radiance values in individual mice revealed some varied responses, with some BA-treated mice showing a downward trajectory at later times (Supplementary Fig. S1c, red colour). However, Kaplan–Meier survival analysis revealed no survival benefits associated with BA treatment in this model (Supplementary Fig. S1d).

**Fig. 2:**
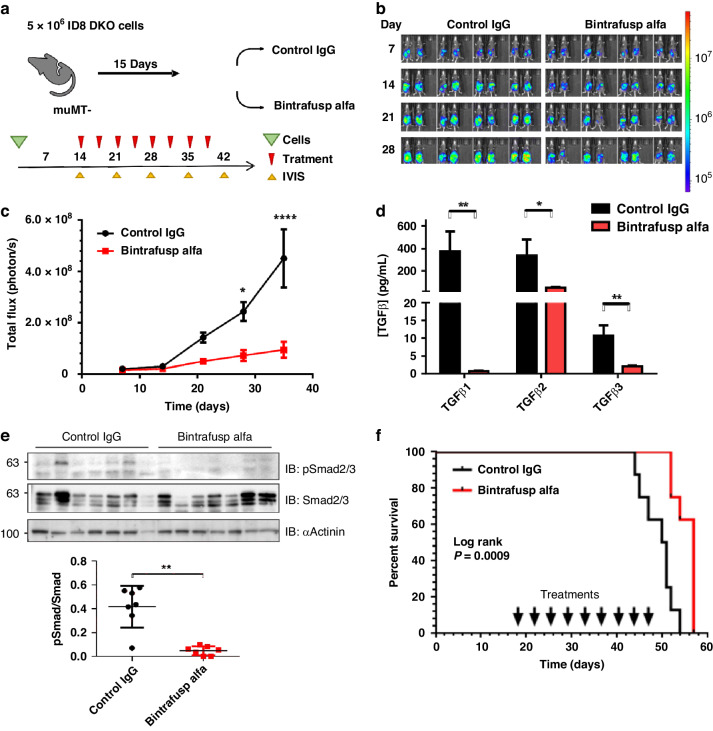
BA improves survival in ID8-derived syngeneic HGSC mouse model. **a** A summarised study design for testing BA in ID8-DKO-Luc/µMT^−^ syngeneic model. **b** Luminescence measurements were obtained through IVIS imaging weekly following the injection of ID8-DKO-Luc cells into the peritoneum of female µMT^−^ mice randomised between control IgG and BA treatments. **c** The graph depicts the quantification of luminescence measurements as total flux (mean ± s.e.m.) at the indicated days post injection (*P < 0.05, ****P < 0.0001 based on two-way ANOVA with multiple comparison testing). **d** Mice were sacrificed at humane endpoints and the ascites was harvested to measure TGF-β ligand concentrations in both groups (*P < 0.05, **P < 0.01 based on ANOVA). **e** Lysates from ascites cells from Control IgG (*n* = 7) or BA (*n* = 7) were subjected to immunoblot (IB) using anti-pSmad2/3, anti-Smad2/3 and anti-α-Actinin antibodies. Representative blots are shown above graph of densitometry values (pSmad/Smad; **P < 0.01 based on paired *T* test). **f** Kaplan–Meier analysis comparing survival of mice treated with control IgG or BA (median survival 50 vs 57 days; log-rank *P* < 0.001).

Since prior study of BA in syngeneic mouse tumour models used B-cell-deficient (µMT^−^) recipient mice to extend BA treatment times while avoiding antibody responses to the humanised protein [30], we revised our HGSC model to use µMT^-^ recipient mice (on a C57BL/6 background; Fig. 2a). The main difference in our study design was a longer treatment window that started on day 14 of this survival study (Fig. 2a, red arrows). Weekly IVIS imaging on individual mice revealed a consistent gradual increase in the radiance signal in the abdomen of mice in the control IgG group compared to the BA-treated group (Fig. 2b). Quantification of the total flux from IVIS imaging revealed that BA-treated mice had significantly lower tumour burden at 28 and 35 days relative to the control IgG (Fig. 2c). As animals reached humane endpoints, we collected ascites fluid, and measured the levels of TGF-β1–3 ligands. Consistent with the TGF-β ligand trap functionality of BA, we observed significant depletion of total TGF-β1, TGF-β2 and TGF-β3 levels in the ascites fluid (Fig. 2d). To examine the impact of BA treatments on TGF-β signalling in this HGSC tumour model, we performed immunoblotting with phosphorylation-specific Smad2/3 (pSmad2/3) or pan-Smad2/3 antibodies on lysates from ascites cells (Fig. 2e; α-Actinin served as a loading control). Consistent with the depletion of TGF-β ligands in the BA treatment group, we observed a significant reduction in Smad2/3 activation compared to control IgG treatments (Fig. 2e, see graph). To measure survival outcomes, Kaplan–Meier curves were generated for control IgG and BA treatment groups, and this revealed a statistically significant extension of survival times with BA treatment compared to control IgG (Fig. 2f).

### BA treatments limited ascites development and inflammatory cytokine production

**Fig. 3:**
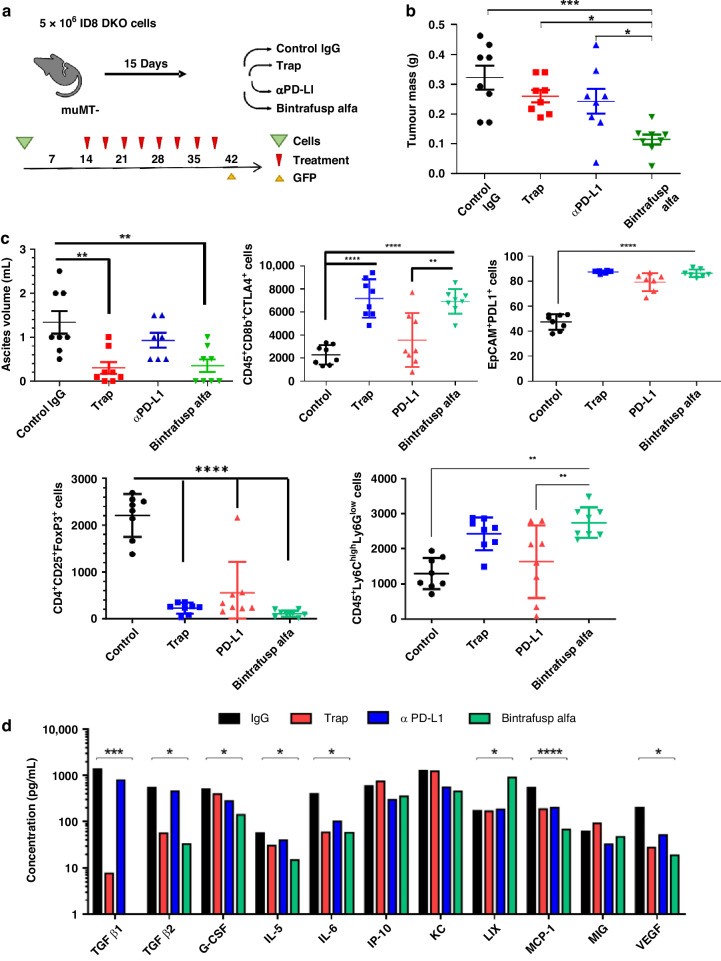
BA impairs tumour growth, ascites development and cytokine composition. An endpoint study was initiated to assess BA treatment effects in ID8-derived HGSC syngeneic model. **a** The study design outlines the injection of ID8-DKO-Luc cells (5 × 10^6^) into female µMT^−^ mice randomised between the indicated treatment groups. At endpoint, ascites and GFP^+^ tumour nodules were isolated for analysis. **b** Mice were sacrificed after 45 days and metastatic tumour nodules were harvested for the recording of tumour mass (*P < 0.05, **P < 0.01, ***P < 0.001 based on ANOVA with multiple comparison testing). **c** Ascites volumes were measured in each treatment group. Cells isolated from ascites samples were subjected to flow cytometry for identification of activated immune cells and tumour cells using antibodies targeting CD45, CD8b, CTLA-4, EpCAM, PD-L1, CD4, CD25, FoxP3, Ly6C and Ly6G (**P < 0.01, ****P < 0.0001 based on ANOVA with multiple comparison testing). **d** Graph depicts results from a Mouse Cytokine 31-Plex used to measure ascites cytokine concentrations from samples in each treatment group (*P < 0.05, ***P < 0.001, ****P < 0.0001 based on ANOVA with multiple comparison testing).

To better characterise the dual functionality of BA during HGSC progression, we performed an endpoint study using the above model, with the addition of TGF-β Trap control (mutations in the antigen-binding portion of the antibody impairs binding to PD-L1) [30], and anti-PD-L1 control treatment groups (Fig. 3a; *n* = 8/group). Based on the control arm of our survival study, we selected day 45 as our endpoint, to allow for sufficient growth of tumour nodules and ascites development for downstream analyses of time-matched samples (Fig. 3a). In this study, we did not use IVIS to measure tumour burden, instead we used the GFP reporter in ID8-DKO-Luc cells to isolate all individual tumour nodules at endpoint for each mouse and determined the overall HGSC tumour mass. Mice treated with BA had significantly less tumour burden when compared to all other treatment arms (Fig. 3b). Interestingly, both BA and the TGF-β Trap control significantly impaired ascites development in this model (Fig. 3c, top left), thus implicating TGF-β as a key contributor to this disease process. To characterise ascite cells, flow cytometry was used to identify cell types and immune checkpoints or activation markers. Consistent with the immune suppressive effects of TGF-β, treatments with TGF-β Trap control or BA led to increased tumour-associated CD8 T cells expressing the activation marker and immune checkpoint CTLA-4 (Fig. 3c). The opposite trend was seen for FoxP3^+^ T regulatory cells, and this was significant with BA and other treatments compared to control (Fig. 3c). All treatments led to increased expression of PD-L1 on EpCAM-expressing HGSC cells in the ascites (Fig. 3c). Further, BA treatments significantly increased the inflammatory monocytes (Ly6C^high^ Ly6G^low^) compared to anti-PD-L1 or control (Fig. 3c). To test how treatments affect cytokines in the tumour microenvironment, we analysed cytokine levels in ascites for each treatment group. As predicted, levels of TGF-β1 and TGF-β2 in the ascites were drastically reduced with BA and TGF-β Trap control treatments (Fig. 3d). BA treatment also resulted in the reduction in cytokines or chemokines known to be critical in HGSC metastasis (G-CSF, IL-5, IL-6 and MCP-1) and ascites development (VEGF; Fig. 3d). While many of these cytokine effects were similar in both BA and TGF-β Trap control treatments compared to control IgG, the observed increase of the chemokine LIX/CXCL5 was unique to BA-treated mice (Fig. 3d), and may contribute to increased inflammatory monocytes we detected (Fig. 3c). These results show that BA treatments protect the animals from development of ascites, likely due to normalising lymph node functions.

### BA treatments altered the tumour immune microenvironment in ID8-derived HGSC tumours

Next, we analysed how BA treatments alter the tumour immune microenvironment (TIME) in the ID8-DKO syngeneic model. We used an immune profiling gene panel to measure differences in gene expression within HGSC tumours from mice treated with either BA or IgG control in the ID8-DKO endpoint study. Using a volcano plot of gene expression and statistical significance thresholds, we identified numerous genes that were differentially expressed in BA-treated tumours compared to control IgG (Supplementary Fig. S2a and Supplementary Tables 1 and 2). As expected, BA-treated tumours had reduced expression of TGF-β target genes (*Thbs1, Vegfa, Runx1*), as well as genes linked to ascites production (*Vegfa*, see Supplementary Table 2 and Supplementary Fig. S2b). BA-treated tumours showed increased expression of genes linked to M1 TAM markers (*Nfkbia*, *C7*) [38, 39], effector memory T cells (*Gzmk*) [40], and activated or exhausted T cells (*Lag3*, *Tigit*; see Supplementary Table 1 and Supplementary Fig. S2c). Since multiple TAM genes were altered at the transcript level, we decided to profile TAMs in HGSC tumour cryosections from the same model, comparing control IgG and BA treatments in ID8-DKO tumours. We used pan TAM marker (CD68) along with M1 marker iNOS, and M2 marker CD206. We detected CD68^+^ TAMs in all tumour sections, with no overt differences in density noted between treatment groups (Supplementary Fig. S3a). However, iNOS^+^ M1 TAMs were found at significantly higher density in sections from BA-treated tumours compared to control (Supplementary Fig. S3a/b). In contrast, CD206^+^ M2 TAMs were significantly lower in BA-treated tumours (Supplementary Fig. S3a/b). Overall, these results are consistent with BA treatments limiting immune suppressive TGF-β signalling and promoting immune activation within the TME.

### BA immunotherapy triggered increased TIME cytotoxicity markers in the BR5/FVB syngeneic model

To extend our studies of BA treatments to an additional HGSC model, we acquired BR5-Luc cells derived from surface ovarian epithelial cells of transgenic Trp53^−/−^:Brca1^−/−^:Tg^−Myc^ mice (FVB strain) [37]. The BR5-Luc cells were responsive to TGF-β treatments and galunisertib treatments (Supplementary Fig. S4a) and expressed inducible PD-L1 in response to IFN-γ (Supplementary Fig. S4b/c). Syngeneic engraftment of BR5-Luc cells injected i.p. into female FVB mice was performed, along with weekly IVIS imaging of D-Luciferin-injected mice. Firstly, we performed a survival study comparing the effects of three treatments with BA or control IgG as depicted (Supplementary Fig. S5a, red arrows). Over 7 weeks, we observed steadily increasing IVIS signal in both groups (Supplementary Fig. S5b). Although BA-treated mice showed a significant reduction in IVIS signal at day 49 (Supplementary Fig. S5c), Kaplan–Meier survival analysis revealed no significant survival benefit with only 3 BA treatments in this model (Supplementary Fig. S5d). This is despite ∼30% of BA-treated mice surviving >2 weeks longer than the control group (Supplementary Fig. S5d).

**Fig. 4:**
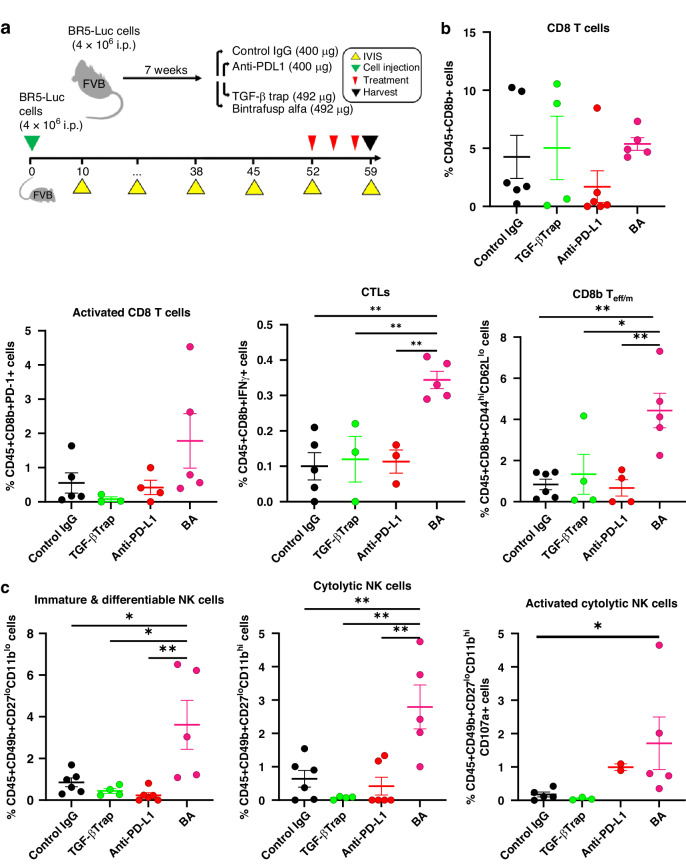
Endpoint study with short-term bintrafusp alfa treatments in BR5 syngeneic model provides evidence of TIME skewing towards T/NK cell activation. **a** The study design outlines the injection of BR5-Luc cells (4 × 10^6^) into female FVB mice randomised between the indicated treatment groups. Treatments were administered three times, 2 days apart during week 7 to avoid neutralising antibodies production that limit efficacy of testing bintrafusp alfa. **b** At endpoint, cells from dissociated tumours were subject to flow cytometry for immunophenotypic analysis of T cells using antibodies targeting surface markers for CD8b, PD-1, CD44, CD62L and intracellular markers against IFN-γ (two mice from Trap control group did not reach endpoint and were removed because they reached protocol-defined endpoint earlier). **c** NK cells were classified using antibodies targeting surface markers for CD49b, CD27, CD11b and CD107a (*P < 0.05, **P < 0.01, ****P < 0.0001 based on ANOVA with multiple comparison testing). Overall, 30,000 lymphocytes were recorded, and data was exported as %parent normalised to %live CD45^+^ immune cells. Gating strategies are shown in Supplementary Fig. S7. CTL cytotoxic T lymphocyte, T_em_ T-effector memory.

Given the encouraging effects of BA treatment on tumour burden (IVIS) in the BR5/FVB model, we decided to repeat the experiment as an endpoint study with the three treatments closer to endpoint, thus allowing TIME alterations to be compared across treatment groups (Fig. 4a). At endpoint, tumours were harvested, dissociated into single-cell suspensions, and stained with surface or intracellular antibody panels designed to assess T or NK cell phenotypes for analysis using flow cytometry. Total CD8 T cells and CD8^+^PD-1^+^ activated T cells trended higher in the BA-treated group compared to controls (Fig. 4b). However, BA-treated tumours had significantly higher cytotoxic T lymphocytes (CTLs; CD8^+^IFN-γ^+^) and CD8 T-effector memory cells (T_em_) compared to other treatment arms (Fig. 4b). Only trending differences in the CD4 compartment were observed in BA-treated tumours (Supplementary Fig. S6a). Next, we tested NK subsets in these tumours and observed a significant increase in immature, cytolytic and activated NK cells with BA treatment compared to controls (Fig. 4c). Other NK subsets were not significantly different between BA and control IgG (Supplementary Fig. S6b). These are promising changes in the TIME with short-term treatments with BA and this may trigger anti-tumour immunity in the longer term.

### BA treatments caused long-term survival in the BR5/FVB syngeneic model of HGSC

**Fig. 5:**
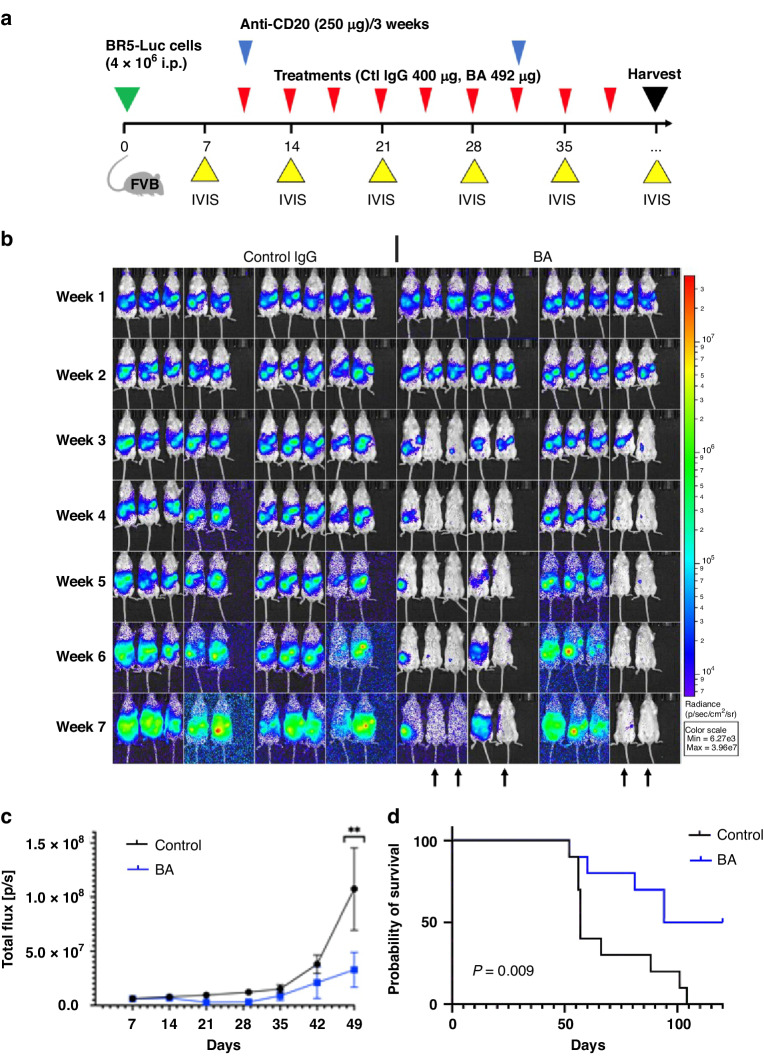
BA promotes survival in BR5/FVB syngeneic HGSC mouse model. **a** A summarised study design for testing BA in BR5-Luc/FVB syngeneic model. **b** Luminescence measurements were obtained through IVIS imaging weekly following the injection of BR5-Luc cells (4 × 10^6^) into the peritoneum of female FVB mice randomised between control IgG or BA treatments. **c** The graph depicts the quantification of luminescence measurements as total flux (mean ± s.e.m.) at the indicated days post injection (**P < 0.01 based on two-way ANOVA with multiple comparison testing). **d** Kaplan–Meier analysis comparing survival of mice treated with control IgG or BA (median survival 57 vs 141.5 days; log-rank *P* < 0.01).

The promising survival outcomes from just three treatments in the immunocompetent BR5/FVB model encouraged us to extend the treatment window, similar to the ID8 survival study. In a follow-up survival study, FVB mice were transiently depleted of B cells using anti-CD20 to avoid neutralisation of the humanised BA and controls and thus extend the treatment window (Fig. 5a, blue arrows). Analysis of the extent of B-cell depletion with flow cytometry revealed that 99% of B cells were depleted in the spleen and peritoneum two weeks after anti-CD20 injection (Supplementary Fig. S7). Weekly IVIS imaging revealed a reduction in HGSC tumour burden in 50% of the BA-treated group compared to control IgG (Fig. 5b). Overall, the BA treatment group had a significant reduction in total flux on day 49 of the survival study compared to the control (Fig. 5c). Upon mice reaching protocol-defined endpoints, Kaplan–Meier survival analysis revealed that BA treatments significantly improved overall survival (Fig. 5d, average 57 days with control vs 141.5 days with BA). In fact, 50% of mice treated with BA survived with undetectable cancer (BA-cured) after all the control mice met their protocol-defined endpoints (Fig. 5d). Although both HGSC models showed survival benefit, the BR5/FVB syngeneic model was more sensitive to BA in terms of tumour regression and long-term survivors.

### BA treatments provided long-term anti-HGSC immunity via expansion of T-effector memory and NK cells

**Fig. 6:**
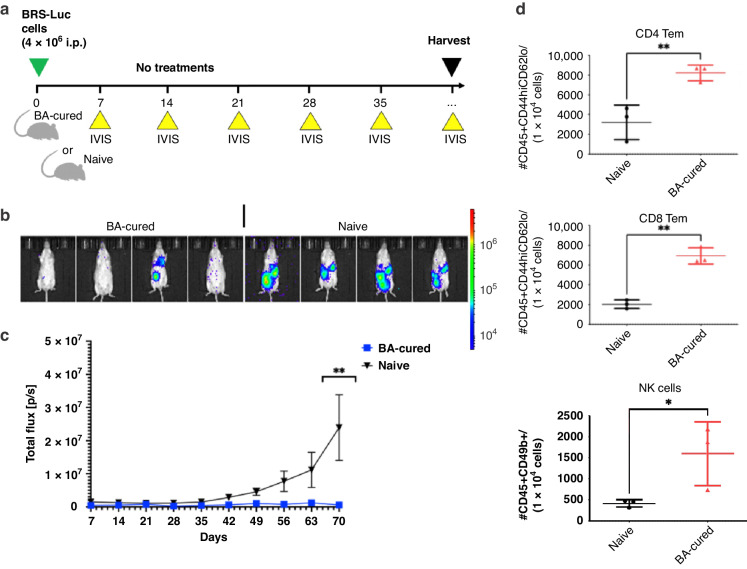
BA promotes long-term protective anti-tumour immunity by improving levels of T-effector memory and NK cells. **a** A summarised re-challenge study design for testing memory responses in BR5-Luc/FVB syngeneic model. **b** Luminescence measurements were obtained through IVIS imaging weekly following the re-injection of BR5-Luc cells (4 × 10^6^) into the peritoneum of cured female FVB mice or treatment-naive mice, without additional treatments. **c** The graph depicts the quantification of luminescence measurements as total flux (mean ± s.e.m.) at the indicated days post injection (**P < 0.01 based on two-way ANOVA with multiple comparison testing). **d** A peritoneal lavage was performed 107 days after the start of re-challenge. Cells from the peritoneum were subjected to flow cytometry for identification of immunological memory and activated immune cells using antibodies targeting CD45, CD8b, CD49b, CD44 and CD62L (*P < 0.05, **P < 0.01 based on ANOVA with multiple comparison testing).

Four of the BA-cured FVB mice from the survival study were subjected to re-challenge with BR5-Luc cells (i.p.) to test for immunological memory responses compared to a control group of age-matched, treatment-naive female FVB mice (Fig. 6a). Without further treatment, weekly IVIS showed that 75% of the BA-cured mice rejected the BR5-Luc cancer cells (Fig. 6b). As a result, BA significantly reduced the total flux of re-challenged survivors compared to the naive control mice (Fig. 6c). To pursue a mechanistic understanding of how BA promotes survival, we considered the major cell types altered by a HGSC vaccine derived from the BR5 model [41]. After 15 weeks of re-challenge, peritoneal lavage, and/or ascites, from naive and BA-cured mice were harvested to perform immunophenotyping by flow cytometry. Our results showed that BA-cured mice had increased populations of CD8 and CD4 T_em_ cell subsets as well as circulating NK cells in the peritoneal cavity compared to naive mice (Fig. 6d). Overall, our results show that in the TGF-β-rich TME of HGSC, immune suppression limits the effects of PD-1/PD-L1 blockade. However, coordinated blockade of TGF-β and PD-L1 signalling by BA treatments can promote long-lasting immune memory responses similar to that acquired by a cancer vaccine.

## Discussion

Cancer immunotherapies with immune checkpoint inhibitors have failed in ovarian cancer patients despite relatively high genome instability and tumour mutational burden [42]. In this study, we show that BA, which simultaneously inhibits PD-L1 and TGF-β, impairs the growth of HGSC tumours and promotes anti-tumour immunity in two models on different genetic backgrounds. In the ID8-derived HGSC mouse model, we demonstrated that BA treatments clear TGF-β from the ascites and lessen the ascites and tumour burden significantly. This correlated with increased activated CD8 T cells in the peritoneum with BA treatments compared to controls, and skewing of the TME towards M1 TAMs. Testing of BA was also extended to the BR5/FVB HGSC mouse model, and we observed improved tumour immunophenotypes at endpoint and significant survival advantages compared to control IgG. Upon re-challenge with BR5 cells, 75% of BA-cured mice were able to reject the tumours without any further treatments. Compared to naive mice, BA-cured mice showed elevated levels of CD4 and CD8 T-effector memory cells and NK cells in the peritoneum. Overall, our study provides evidence that coordinated inhibition of PD-L1 and TGF-β can provide improved control of HGSC progression, and in some cases lead to protective anti-tumour immunity.

Our paper builds on prior studies of ICIs in syngeneic HGSC models. Prior studies have implicated immune exhaustion of TILs and PD-L1 expression on MDSCs in the ID8 model, and that this could be overcome with an ID8-derived vaccine followed by PD-1 or PD-L1 blockade [43]. More recently, a vaccine prepared from cryogenic silicified BR5 cells was shown to be effective in both preventative and therapeutic treatment regimes in the BR5/FVB model [41]. In our study, BA treatments were more effective in the BR5/FVB syngeneic model, with 50% survivors that were immune to re-challenge with BR5 cells. Similar to the vaccine approach by Guo et al. [41], we detected increased T-effector memory and NK cells in the peritoneum of these BA-cured mice compared to naive mice. This suggests more active patrolling of immune cells can be induced following BA treatments in HGSC models, leading to either a fully functional memory response (BR5 model) or a partial response that slowed tumour progression and reduced ascites development (ID8-DKO model). Further studies will be needed to identify biomarkers of durable responses to BA that can inform future studies aimed at translation of these findings into new treatments for HGSC. In colorectal cancer models, TGF-β was shown to be a major driver of immune exclusion and evasion that could be reversed by the TGFBR1 inhibitor galunisertib in combination with PD-L1 blockade [44]. Together, these studies support the benefits of combining TGF-β inhibitors with immune checkpoint inhibitors to drive effective immunotherapy responses in mouse cancer models.

With relevance to ovarian cancer, several recent preclinical studies have tested BA in combination treatment regimes. Of interest, BA prolonged survival and reversed lung fibrosis when combined with localised radiotherapy in several poorly immunogenic murine tumour models that were previously resistant to radiation [45]. These results were further enhanced when BA was combined with DNA-dependent protein kinase inhibitor peposertib [46]. This suggests potential for investigating the benefits of radioimmunotherapy combinations in HGSC, perhaps targeting focal tumours in the peritoneum.

BA has also undergone several clinical trials for human patients in a number of cancer types, with evidence of manageable safety profile and some responses observed [47]. In heavily pretreated lung cancer patients, a recent Phase I study reported partial responses or stable disease in 15% of patients [48]. However, in a larger Phase III trial of BA versus pembrolizumab in treatment-naive patients with PD-L1-high advanced lung cancer, BA was not superior to pembrolizumab leading to early termination of the trial [49]. Further stratification of patients with biomarkers indicative of high TGF-β signalling in the TIME, such as Thrombospondin-1, may help identify patients most likely to benefit from the dual activities of BA. Some clinical activity of BA was also observed in human papillomavirus-positive head and neck cancers [33]. As a neoadjuvant immunotherapy for head and neck cancer, BA treatments reduced the tumour retention of exhausted CD103^+^ CTLs in responders which may explain improved systemic anti-tumour immunity in these patients [50]. However, in HGSC the presence of CD103^+^ has been linked to improved prognosis [51, 52]. It will be important to define the biomarkers of response to BA in HGSC to stratify patients most likely to respond to BA in future trials for ovarian cancer.

A limitation of our study involved the manipulation of B cells in some of our HGSC models to avoid the production of neutralising antibodies that develop against the humanised BA protein [31]. For this reason, BA treatments in B-cell competent models are limited to 6 days to prevent neutralisation leading to an incomplete assessment of BA [31]. We made similar observations regarding the impact of BA on survival in both HGSC models with a need for extended treatments. In ovarian cancer, B cells produce IgG antibodies that target tumour antigens while situated in stromal tertiary lymphoid structures (TLS) [53]. Recently, TLS have been recognised as critical effectors of anti-tumour immunity, with the presence of TLS correlated with greater B-cell infiltration and response to ICI in patients with melanoma [54]. Several reviews have summarised the ability of B cells to be both positive and negative prognostic indicators in ovarian cancer [55]. Future studies using other genetic or pharmacological approaches will allow for a more complete understanding of the benefits of coordinated inhibition of PD-L1 and TGF-β in HGSC.

In addition to BA, other dual inhibitors of PD-L1 and TGF-β have been developed. This includes a bispecific antibody targeting human TGF-β and PD-L1 (BiTP) and a mouse-reactive version YM101. In triple-negative breast cancer models, BiTP treatments decreased collagen deposition and increased tumour CTLs, leading to superior anti-tumour activity compared to parental antibodies [56]. Given our results with BA treatments of HGSC models, it would be interesting to test YM101 treatments to co-target TGF-β and PD-L1 in these models. In this study, we show that BA treatments enhance survival by limiting HGSC tumour growth relative to control treatments. Both BA and the TGF-β Trap control treatments led to reduced levels of TGF-β1 and TGF-β2 in ascites as well as VEGF, thus validating previous studies implicating TGF-β signalling in the development of ascites [24, 57]. Interestingly, BA modified the levels of several key cytokines within the ascites. MCP-1 is secreted by omental adipocytes in ovarian cancer and orchestrates the metastatic dissemination of tumour cells to the omentum [58]. The reduced levels of MCP-1 in the BA-treated mice partially explain the dramatic reduction in metastatic tumour burden observed at the endpoint since most metastatic nodules were identified in the omentum regardless of treatment. Furthermore, G-CSF and IL-6, reduced in BA ascites, contribute to the motility and invasion of HGSC cells [59–62]. VEGF is also associated with malignant ascites and metastasis in human and mouse ovarian cancer [63, 64]. We have shown that BA orchestrates a broad reversal of factors that contribute to HGSC disease progression.

To build on our mechanistic understanding of the effects of BA in the ID8/µMT^-^ model, we compared differentially expressed genes from tumours treated with BA versus IgG control. The reduced expression of murine M2 TAM marker Chil3 [65] observed with BA treatment in our model provided the rationale for validating macrophage skewing, which we showed in our HGSC tumours using M1 and M2 TAM markers. Likewise, increased expression of NF-κB target gene NFkbia [38] is consistent with BA treatments inducing M1-like classically activated macrophages with inflammatory functions and increased Complement-7 (C7) production in tumours [39]. These findings suggest that BA treatments in HGSC skew macrophage polarisation towards an M1 phenotype. Also, BA treatments increased the levels of a T-effector memory marker Granzyme K (Gzmk), previously reported to involve neutrophil-T cell interactions in colorectal cancer [40]. In the ID8-derived HGSC model, BA treatments also increased the expression of secondary immune checkpoints Lag3 and Tigit, and it will be interesting to determine if blockade of these checkpoints may increase BA response rates in our HGSC models.

The mechanisms leading some HGSC-bearing mice to be cured by BA treatments in our study will be important to fully characterise and maximise the potential clinical translation. At the cellular level, BA-treated HGSC tumours skewed towards increased M1 TAMs, active CTLs, and expansion of T-effector memory cell populations. Some of these results are consistent with effects observed with BA treatments in colon and breast syngeneic models [29]. It is worth noting that in our study of BA-cured mice, we detected increased peritoneal NK cells compared to naive mice, and a number of NK subsets were altered by BA in our BR5 endpoint study (tumour-infiltrating immature NK cells and cytolytic NK cells expressing CD107a). We employed an NK cell gating strategy to classify NK cell subsets based on maturation and function [66], and shown to be regulated by TGF-β [67]. In ovarian cancer, cytolytic NK cells exposed to TGF-β in the TME are suppressed and acquire similar phenotypes to those in deciduae that facilitate angiogenic and tolerogenic functions [68]. Along with inducing suppressive effects from Tregs and TAMs in the TME, TGF-β is known to downregulate NK cell activating receptors NKG2D and NKp30 and skew development towards type 1 innate lymphoid cells to facilitate immune escape [69, 70]. Impaired release of cytolytic effectors such as granules containing perforin and granzyme B and IFN-γ have been reported for NK cells isolated from patients with HGSC with low NKp30 [71]. Thus, BA treatments may overcome impaired NK cell functions in HGSC. Overall, our data reveals evidence of a coordinated anti-tumour immune response by NK cells and CD8 T cells in vivo only when both PD-L1 and TGF-β pathways are blocked by BA. In our HGSC models, BA treatments appear to expand immature and cytolytic NK cell subsets, compared to other treatment groups. This aligns with the recent consideration of NK cell therapy as a promising direction with ample potential in ovarian cancer [72].

In conclusion, this paper provides evidence of promising anti-tumour immunity induced by BA treatments in HGSC syngeneic models derived from distinct genetic backgrounds. Localisation of BA to the PD-L1-positive TME allows for precision targeting of the immune suppressive TGF-β within TIME that limits immune surveillance and developing memory responses. Our results in preclinical ovarian cancer models support further testing of BA in clinical trials for HGSC patients with potential stratification of patients having high TGF-β in ascites or circulating biomarkers related to TGF-β.

### Supplementary information

Supplemental materials

## Data Availability

The datasets used and/or analysed during the present study are available from the corresponding author on reasonable request.
