# Supplementary material for: Blockade of TGF-β and PD-L1 by bintrafusp alfa promotes survival in preclinical ovarian cancer models by promoting T effector and NK cell responses
Source: Br J Cancer. 2024 Apr 15;130(12):2003–15. doi: 10.1038/s41416-024-02677-9 (PMC11183086; doi:10.1038/s41416-024-02677-9)
Supplement: Supplementary file 1 — Supplemental materials [file 41416_2024_2677_MOESM1_ESM.pdf]

**Blockade of TGF-β and PD-L1 by bintrafusp alfa promotes survival in preclinical ovarian cancer models by promoting T effector and NK cell responses**

Jacob Kment,[1,2,4] Dan Newsted,[1,2,4] Stephanie Young,[1] Michael Vermeulen,[1] Brian Laight,1,3 Peter Greer,1,3 Yan Lan,[4¥] and Andrew W Craig [1,2*]

[1] Cancer Biology & Genetics division, Queen's Cancer Research Institute, Kingston ON K7L 3N6, Canada; [2] Department of Biomedical and Molecular Sciences, Queen's University, Kingston ON K7L 3N6, Canada; [3] Department of Pathology and Molecular Medicine, Queen's University, Kingston ON K7L 3N6, Canada; [4] EMD Serono Research & Development Institute, Inc., Billerica, MA 01821 USA, an affiliate of Merck KGaA;

[4] These authors contributed equally: Jacob Kment and Dan Newsted

¥ employed during time of the study

**SUPPLEMENTARY MATERIALS**

uncropped blots for Fig 1A

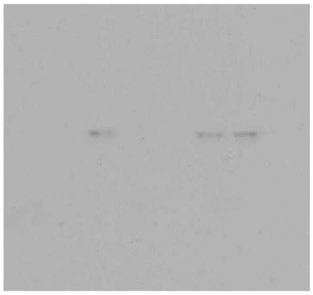

pSmad2/3

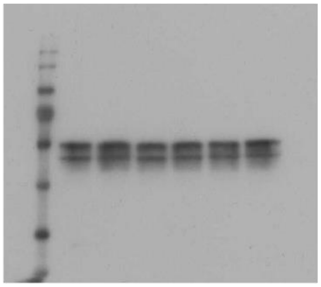

Smad2/3

uncropped blots for Fig 2E

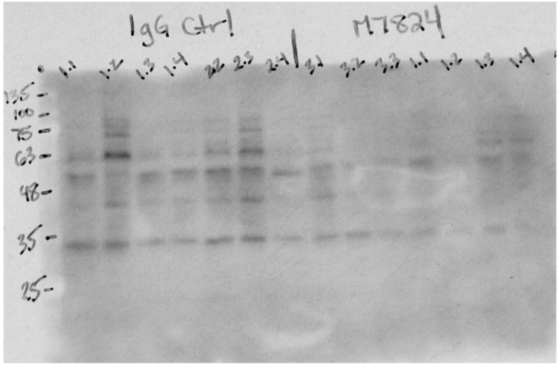


pSmad2/3

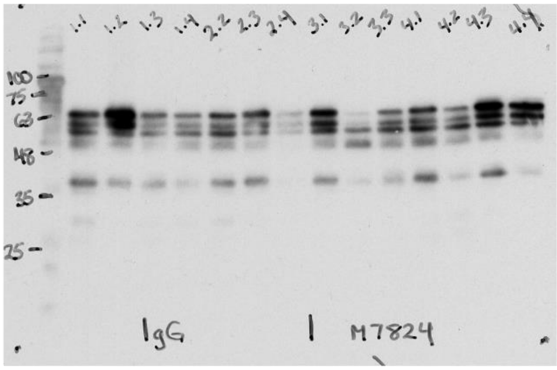


Smad2/3

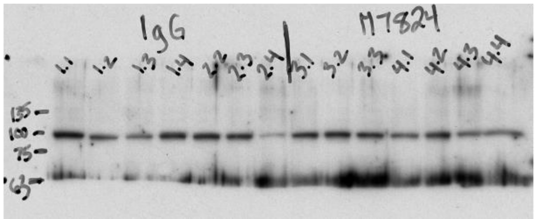


α-Actinin

# SUPPLEMENTARY TABLES

## Supplementary Table 1. Significantly upregulated genes in BA vs IgG-treated HGSC tumors

| Gene name | Log2 FC | P-value | Pathways |
|---|---|---|---|
| Tnfsf10 | 0.678 | 0.00108 | Apoptosis, CD molecules, Cytokines & Receptors, TNF Superfamily |
| C7 | 5.86 | 0.0016 | Complement Pathway, Innate |
| Lag3 | 1.07 | 0.0031 | CD molecules, Interleukins, MHC, NK Cell Functions, T-Cell Functions |
| Ccl3 | 1.36 | 0.00617 | Adaptive, Chemokines & Receptors, Humoral, Inflammation, NK & T-Cell Functions |
| Tab1 | 0.245 | 0.00634 | Basic Cell Functions, Innate |
| Cxcl10 | 0.741 | 0.00724 | Adaptive, Chemokines & Receptors, Inflammation, Innate, Leukocyte Functions |
| Ccl24 | 1.16 | 0.0113 | Adaptive, Chemokines & Receptors, Cytokines & Receptors, Inflammation |
| Lyve1 | 2.29 | 0.013 | Adhesion, Transporter Functions |
| Cxcl11 | 1.04 | 0.0148 | Chemokines & Receptors, Cytokines & Receptors, Inflammation, Innate |
| Irak2 | 0.405 | 0.0169 | Cytokines & Receptors, Innate, Interleukins, TLR |
| Irf2 | 0.304 | 0.0185 | Adhesion, Innate Immunity |
| Relb | 0.149 | 0.0215 | Antigen Processing, Dendritic Cell Functions, T-Cell Functions |
| Tigit | 1.49 | 0.0217 | Interleukins, T-Cell Functions |
| Cd160 | 0.989 | 0.0218 | CD molecules, MHC |
| Il15ra | 1.69 | 0.0222 | CD molecules, NK Cell Functions |
| Gzma | 1.2 | 0.023 | Apoptosis |
| Tnfrsf11a | 0.638 | 0.0241 | Adaptive, CD molecules, Cytokines & Receptors, Interleukins, TNF Superfamily |
| Cfb | 0.8 | 0.0242 | Complement Pathway, Innate |
| Tbk1 | 0.305 | 0.0249 | Innate, Interferon, Pathogen Response, TLR |
| Cd38 | 0.622 | 0.0259 | Apoptosis, B-Cell Functions, CD molecules |
| Gzmk | 3.52 | 0.0267 | Innate Immunity, Memory T cells |
| Klrc1 | 1.02 | 0.0269 | CD molecules, NK Cell Functions |
| Il11ra1 | 0.839 | 0.0304 | Cytokines & Receptors, Interleukins, NK Cell Functions |
| Nfkbia | 0.343 | 0.0309 | Innate, Macrophage Functions, Pathogen Response, TLR |
| Tgfbr2 | 0.258 | 0.0319 | Apoptosis, Cancer Progression |
| Serping1 | 0.467 | 0.033 | Complement Pathway, Innate |
| Cd47 | 0.169 | 0.0349 | CD molecules, Inflammation, T-Cell Functions, Transporter Functions |
| Igf1r | 0.378 | 0.0389 | CD molecules, Cytokines & Receptors, Senescence |
| Igf2r | 0.311 | 0.0393 | Apoptosis, CD molecules, Transporter Functions |
| Irak4 | 0.14 | 0.0401 | Cytokines & Receptors, Innate, Interleukins |
| Klrg1 | 0.902 | 0.0409 | Innate |
| Cd | 0.829 | 0.0413 | CD molecules, NK Cell Functions |
| Ddx58 | 0.263 | 0.0483 | Innate, Interferon |
| Mertk | 0.82 | 0.0494 | Adhesion, Apoptosis, NK Cell Functions, Transporter Functions |
| Egr1 | 0.703 | 0.0496 | Interleukins, Senescence, T-Cell Functions |

## Supplementary Table 2. Significantly downregulated genes in BA vs IgG-treated HGSC tumors

| Gene name | Log2 FC | P-value | Pathways |
|---|---|---|---|
| Ncam1 | -2.46 | 0.000411 | Adhesion, CD molecules, NK Cell Functions |
| Vegfa | -1.13 | 0.0046 | Apoptosis, Cytokines & Receptors, Macrophage Functions, T-Cell Functions |
| Sh2b2 | -0.878 | 0.00539 | B-Cell Functions, Cytokines & Receptors |
| Cspg4 | -0.78 | 0.00765 | Cancer Progression, Inflammation |
| Cd44 | -0.71 | 0.00789 | Cancer Progression, Cytokines & Receptors, Transporter Functions |
| Pdgfc | -1.04 | 0.00845 | Basic Cell Functions |
| Runx1 | -0.671 | 0.00897 | TGF-β pathway, Hematopoiesis |
| Ccl17 | -2.25 | 0.009 | Chemokines & Receptors, Cytokines & Receptors, Inflammation, Innate |
| Il2ra | -1.68 | 0.0177 | Adaptive, B-Cell Functions, CD molecules, Inflammation, T-Cell Functions |
| Thbs1 | -0.62 | 0.0187 | Adaptive, Cell Cycle, Inflammation, Macrophage Functions |
| Socs3 | -0.486 | 0.0222 | T-Cell Functions |
| Clu | -0.413 | 0.0226 | Apoptosis, Cancer Progression, Innate |
| Jun | -0.331 | 0.0247 | Apoptosis, Pathogen Response |
| Trem1 | -0.945 | 0.026 | CD molecules, Humoral |
| Rel | -0.205 | 0.0263 | Cytokines & Receptors, Interleukins, T-Cell Functions |
| Cxcl13 | -2.3 | 0.027 | Adaptive, B-Cell Functions, Chemokines & Receptors, Inflammation, T-Cell Functions |
| C1s1 | -0.514 | 0.0299 | Complement Pathway, Innate |
| Cx3cl1 | -0.944 | 0.0302 | Chemokines & Receptors, Cytokines & Receptors, Macrophage Functions |
| Ripk2 | -0.401 | 0.0306 | Inflammation, Innate, Pathogen Response, T-Cell Functions |
| Yy1 | -0.152 | 0.0316 | T-Cell Functions |
| Ccnd3 | -0.233 | 0.0319 | Cell Cycle, T-Cell Functions |
| Hif1a | -0.276 | 0.0322 | Apoptosis, Cancer Progression |
| Chil3 | -1.67 | 0.0337 | Basic Cell Functions |
| Ccl1 | -1.45 | 0.034 | Adaptive, Chemokines & Receptors, Cytokines & Receptors, Inflammation |
| Pvr | -0.283 | 0.0359 | CD molecules, NK Cell Functions, T-Cell Functions |
| Spp1 | -0.867 | 0.037 | Adhesion, Apoptosis, Cytokines & Receptors, T-Cell Functions |
| Ccr6 | -1.49 | 0.037 | Adaptive, CD molecules, Chemokines & Receptors, Humoral, Innate, T-Cell Functions |
| Tnfsf8 | -1.26 | 0.0426 | CD molecules, Cytokines & Receptors, T-Cell Functions, TNF Superfamily |
| Il1a | -1.39 | 0.0464 | Adaptive, Cytokines & Receptors, Inflammation, Innate, Interleukins |
| Itch | -0.0614 | 0.0472 | Chemokines & Receptors, Innate, T-Cell Functions |

**SUPPLEMENTARY FIGURES**

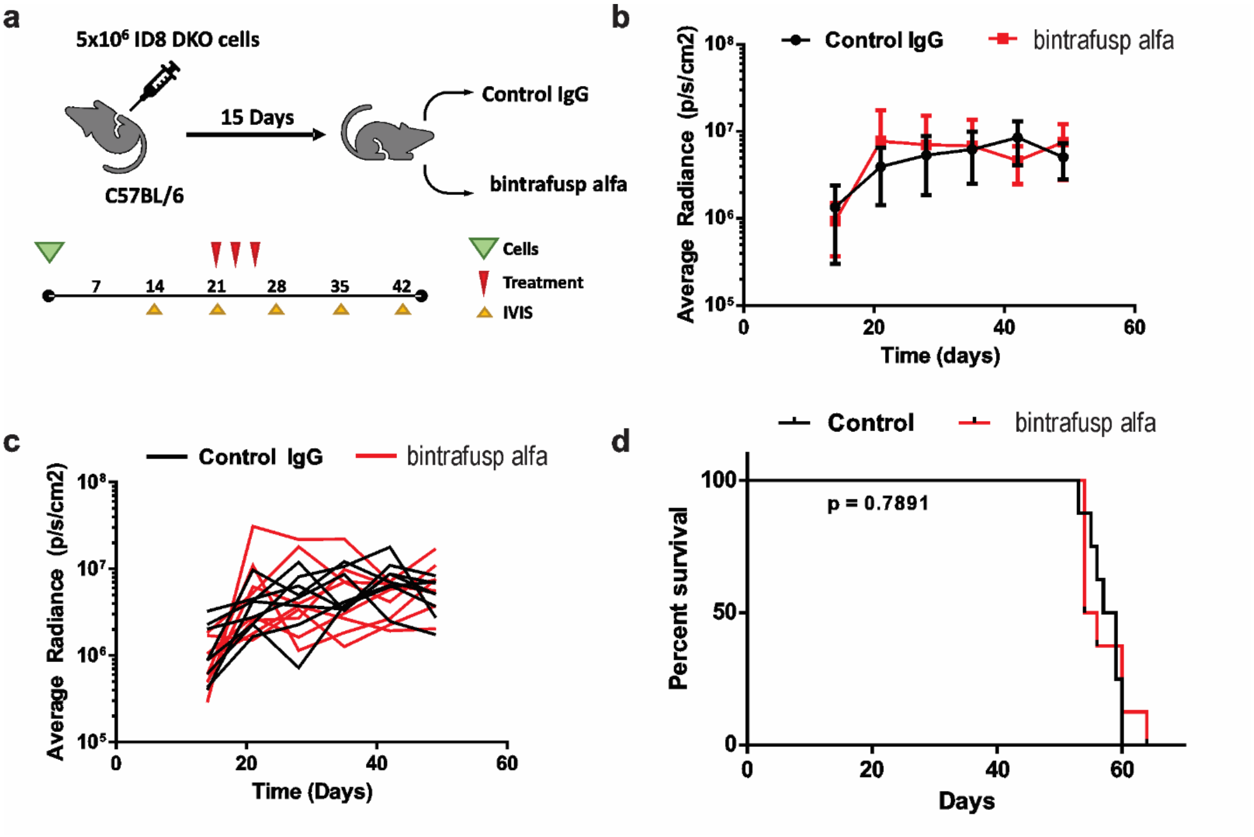


**Fig. S1 BA treatments during week 3 failed to improve survival in HGSC tumor-bearing C57BL/6 mice.**

**a** ID8-DKO-Luc cells were injected into the peritoneum of female C57BL/6 mice. After 15 days, weekly IVIS imaging was performed to measure luminescence as a readout for HGSC tumor burden as described in Materials and Methods. On days 21, 23 and 25, mice received i.p. injections of BA (492 µg) or a control IgG (400 µg; n=7/group). **b** Graph depicts luminescence quantification as average radiance for each treatment group over the indicated time points. **c** Graph depicts average radiance values per mouse in the study, with control group (black lines), and BA group (red lines). **d** Kaplan-Meier survival curve was generated for animals that reached animal protocol-defined humane endpoints.

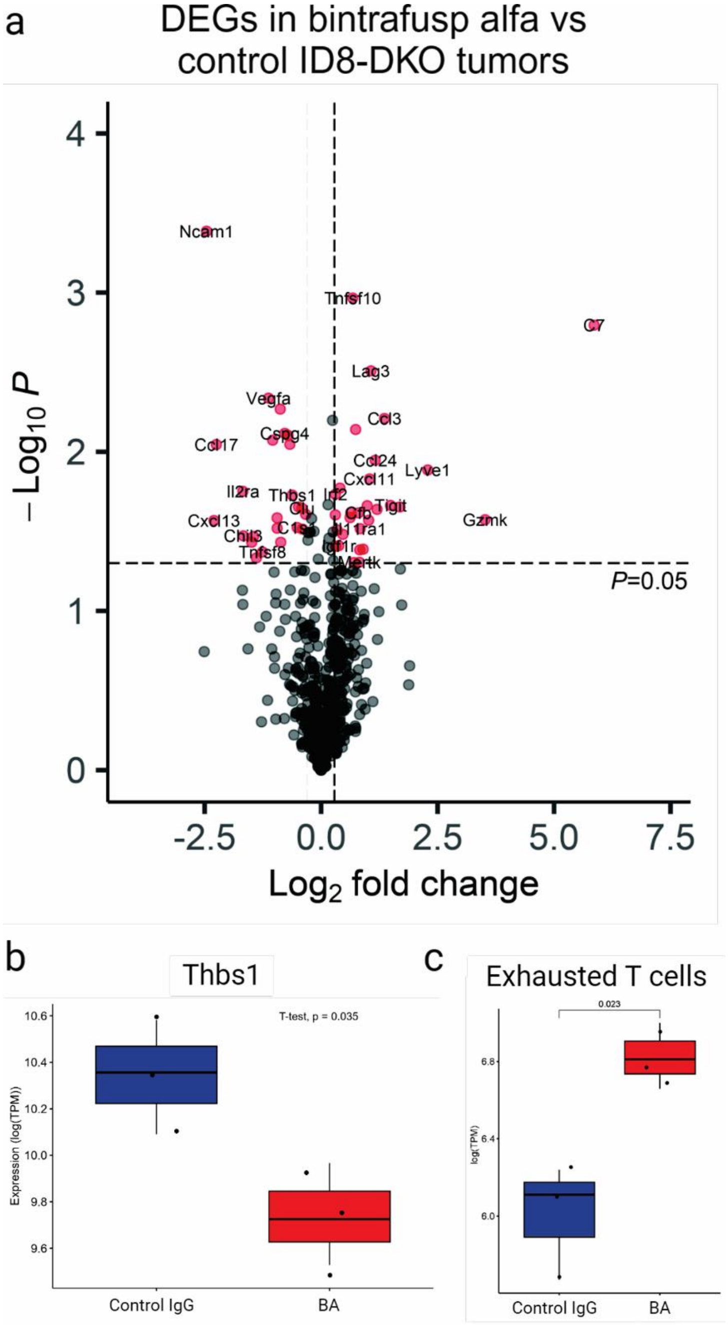


**Fig. S2 Altered gene expression patterns in metastatic tumor nodules treated with BA.**

RNA isolated from metastatic ID8-DKO tumor nodules from the indicated treatment groups was obtained at endpoint and subjected to gene expression analysis using the Nanostring PanCancer Immune Profiling Gene Expression Panel. Analysis of DEGs was performed using nSolver software (Nanostring). **a** A volcano plot of DEGs in BA-treated tumors compared to tumors treated with Control IgG is shown (* $p<0.05$, ** $p<0.01$, *** $p<0.001$ based on ANOVA with multiple comparison testing). **b/c** Box plots are shown comparing Thbs1 or exhausted T cell signatures between control IgG and BA ($p<0.05$ based on unpaired T-test).

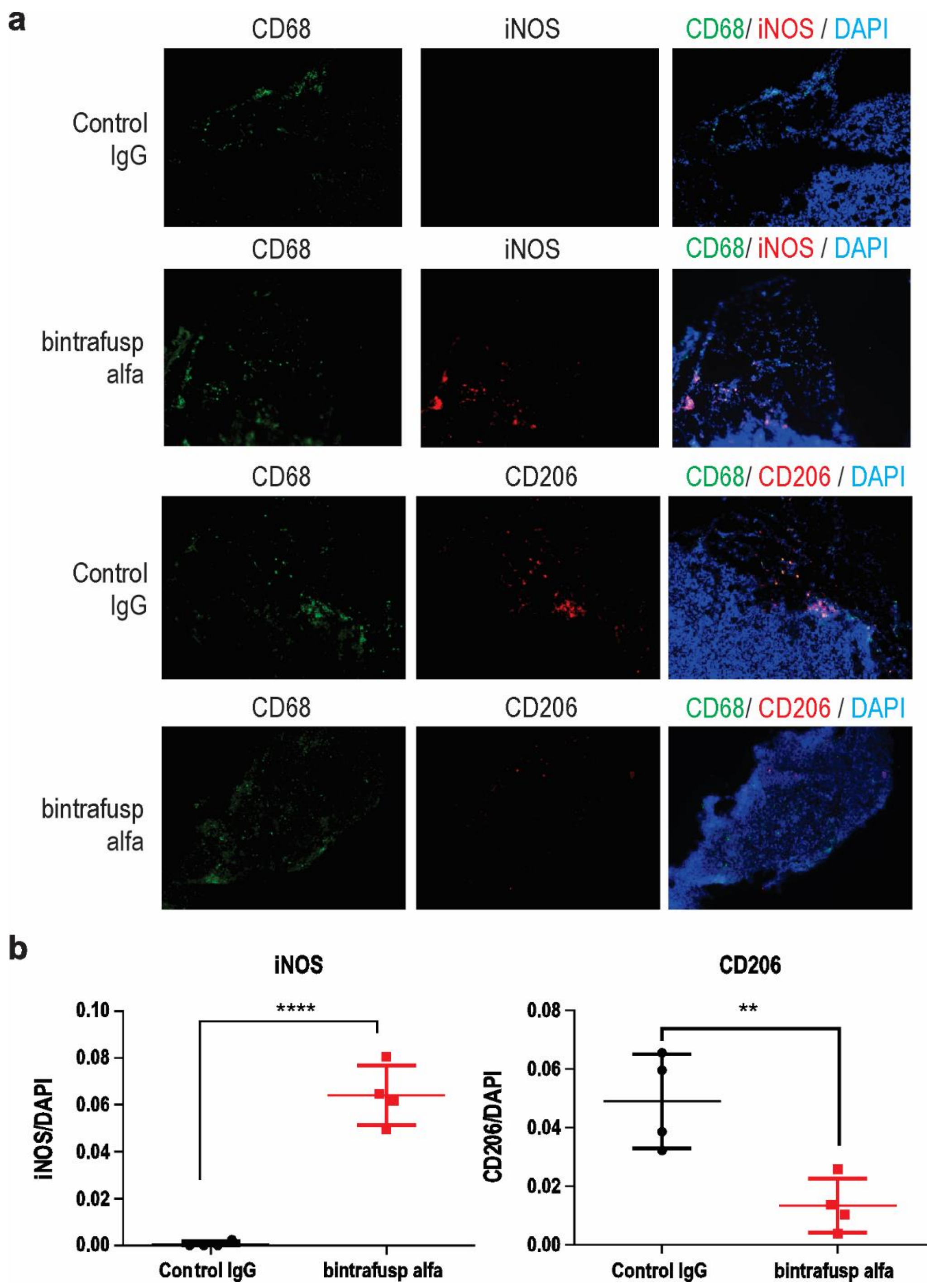


**Fig. S3 BA promotes skewing of tumor-associated macrophage towards M1.**

**a** Cryosections from ID8-DKO HGSC tumor nodules from Control IgG- or BA-treated mice were stained with AlexaFluor (AF)-conjugated anti-CD68 (AF488) paired with either anti-iNOS (AF594) or anti-CD206 (AF594) and DAPI to visualize tumor area. Representative epifluorescence images are shown for individual channels and overlay with DAPI. **b** Graphs indicate the ratios of iNOS-positive or CD206-positive area relative to DAPI-positive areas for individual tumors from separate mice (n=4/group; n=12-16 fields/mouse; ** $p<0.01$, **** $p<0.0001$ based on unpaired T-test).

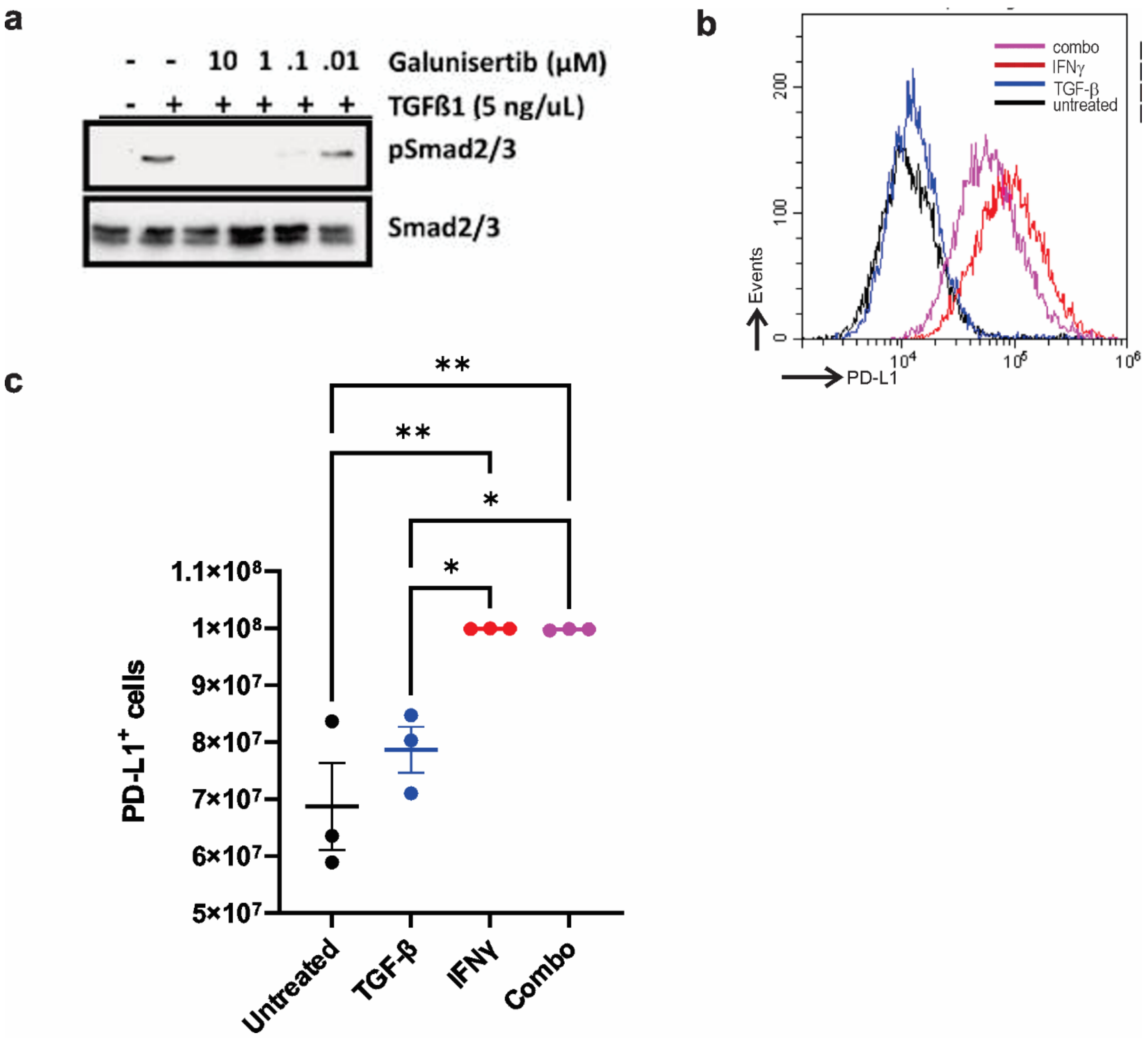


**Fig. S4 Characterization of the syngeneic BR5-Luc mouse HGSC for testing of BA.**

**a** Starved BR5-Luc cells were co-treated with TGF-β1 (5 ng/uL) and increasing doses of TGFBR1 inhibitor LY2157299/Galunisertib (0.1-10 µM) for one hour and Smad2/3 phosphorylation was compared using immunoblotting. **b** BR5-Luc cells were stimulated with TGF-β1 (5 ng/uL) or IFN-γ (20 ng/uL) for 72 hours and surface expression of PD-L1 was compared using flow cytometry. **c** The graph depicts a quantification of the percentage of cells expressing PD-L1 (* $p<0.05$ based on unpaired T-test).

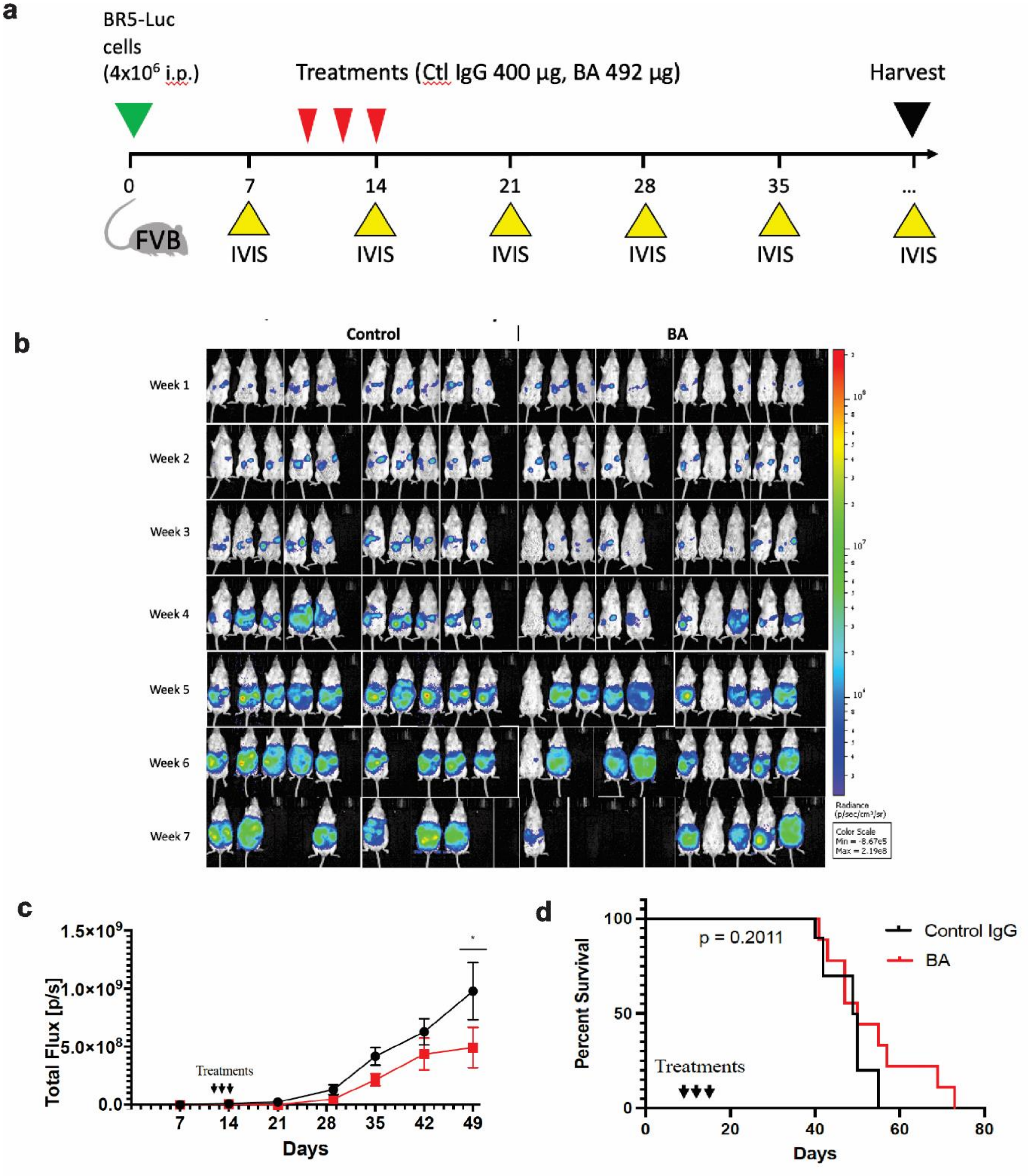


**Fig. S5 Limited treatment window with BA showed promising effects in immunocompetent FVB mice.**

**a** BR5-Luc cells were injected into the peritoneum of female FVB mice. **b** After 7 days, weekly IVIS imaging was performed to measure luminescence as a readout for HGSC tumor burden as described in Materials and Methods. On days 11, 33 and 15, mice received i.p. injections of BA (492 µg) or a control IgG (400 µg; n=10/group). **c** Graph depicts luminescence quantification as total flux for each treatment group over the indicated time points (* $p<0.05$). **d** Kaplan-Meier survival curve was generated for animals that reached animal protocol-defined humane endpoints.

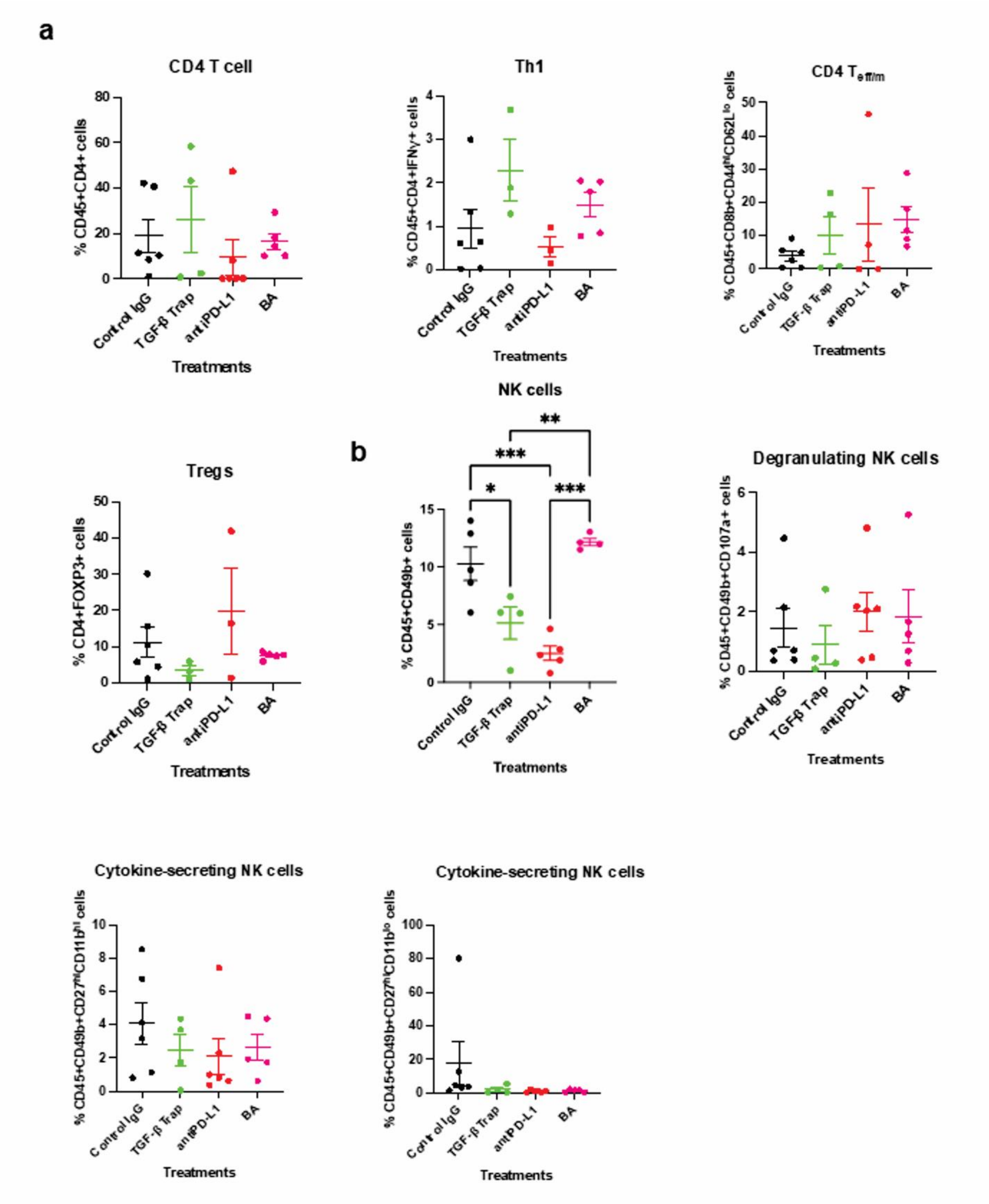


**Fig. S6 Assessment of TIME with short term bintrafusp alfa treatments in BR5 syngenieic model.**

BR5-Luc cells ($4x10^6$) were injected into female FVB mice randomized between Control IgG (400 µg), TGF-β Trap (492 µg), anti-PD-L1 (400 µg) or BA (492 µg). Treatments were administered 3 times, 2 days apart during week 7 to avoid neutralizing antibodies produced by B cells that limit efficacy of testing bintrafusp alfa. At endpoint, cells from dissociated tumors were subject to flow cytometry for immunophenotypic analysis of **a** T cells using antibodies targeting CD4, CD44, CD62L, along with intracellular markers for IFN-γ and FOXP3. **b** NK cells were classified using antibodies targeting surface markers for CD49b, CD107a, CD27 and CD11b (* $p<0.05$, ** $p<0.01$, *** $p<0.001$ based on ANOVA with multiple comparison testing). Overall, 30,000 lymphocytes were recorded, and data was exported as %parent normalized to %live $CD45^+$ immune cells (see S7 for gating strategies).

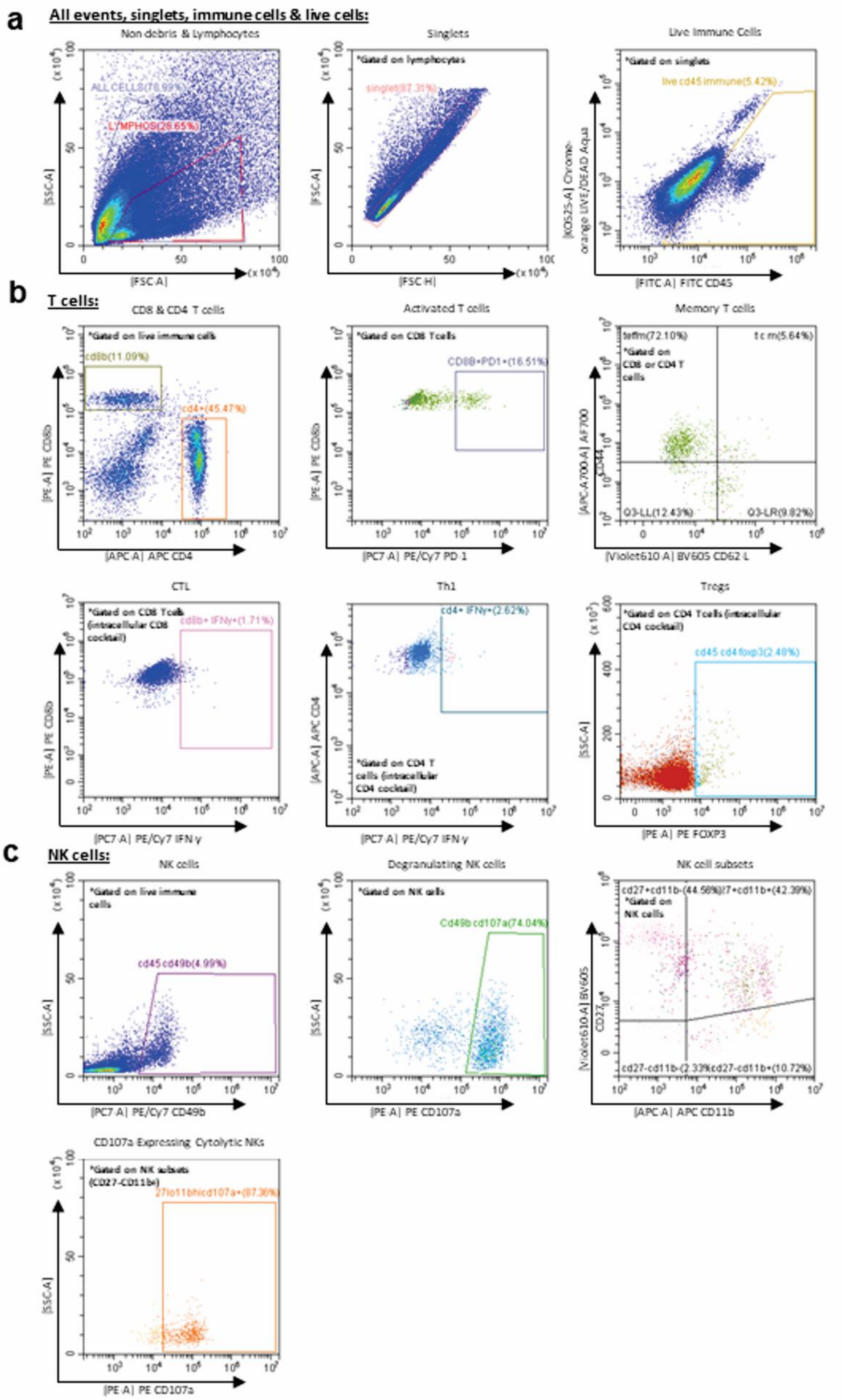
a
All events, singlets, immune cells & live cells:
Non debris & Lymphocytes
ALL CELLS(78.99%)
LYMPHOS(28.65%)
|SSC-A|
|FSC-A|
Singlets
*Gated on lymphocytes
singlet(87.31%)
|FSC-A|
|FSC-H|
Live Immune Cells
*Gated on singlets
live cd45 immune(5.42%)
|KO525-A| Chrome-orange LIVE/DEAD Aqua
|FITC-A| FITC CD45
b
T cells:
CD8 & CD4 T cells
*Gated on live immune cells
cd8b(11.09%)
cd4+(45.47%)
|PE-A| PE CD8b
|APC-A| APC CD4
Activated T cells
*Gated on CD8 Tcells
CD8B+PD1+(16.51%)
|PE-A| PE CD8b
|PC7-A| PE/Cy7 PD-1
Memory T cells
teffm(72.10%)
t c m(5.64%)
*Gated on CD8 or CD4 T cells
Q3-LL(12.43%)
Q3-LR(9.82%)
|APC-A700-A| AF700 CD44
|Violet610-A| BV605 CD62-L
CTL
*Gated on CD8 Tcells (intracellular CD8 cocktail)
cd8b+ IFNγ+(1.71%)
|PE-A| PE CD8b
|PC7-A| PE/Cy7 IFN γ
Th1
cd4+ IFNγ+(2.62%)
*Gated on CD4 T cells (intracellular CD4 cocktail)
|APC-A| APC CD4
|PC7-A| PE/Cy7 IFN γ
Tregs
*Gated on CD4 Tcells (intracellular CD4 cocktail)
cd45 cd4 foxp3(2.48%)
|SSC-A|
|PE-A| PE FOXP3
c
NK cells:
NK cells
*Gated on live immune cells
cd45 cd49b(4.99%)
|SSC-A|
|PC7-A| PE/Cy7 CD49b
Degranulating NK cells
*Gated on NK cells
Cd49b cd107a(74.04%)
|SSC-A|
|PE-A| PE CD107a
NK cell subsets
cd27+cd11b-(44.56%)
27+cd11b+(42.39%)
*Gated on NK cells
cd27-cd11b-(2.33%)
cd27-cd11b+(10.72%)
|Violet610-A| BV605 CD27
|APC-A| APC CD11b
CD107a Expressing Cytolytic NKs
*Gated on NK subsets (CD27-CD11b+)
27lo11bhicd107a+(87.36%)
|SSC-A|
|PE-A| PE CD107a

**Fig. S7 Representative flow cytometry gating strategies using antibodies targeting surface and intracellular antigens on T/NK cells isolated from dissociated tumors in the BR5-Luc endpoint study.**

At endpoint, tumors were mechanically digested prior to staining with flow cytometry. **a** Representative gates for non-debris/lymphocytes, singlets and live $CD45^+$ immune cells were applied as baseline gates for all antibody cocktails. **b** $CD8b^+$ T cells were further classified with antibodies targeting surface markers for PD-1, CD44, CD62L, and IFN-γ in a separate intracellular cocktail. Similarly, $CD4^+$ T cells were further classified with antibodies targeting surface antigens for CD44, CD62L, along with FOXP3 and IFN-γ in separate intracellular cocktails. **c** Last, $CD49b^+$ NK cells were further classified with antibodies targeting surface markers for CD27, CD11b and CD107a. Overall, 30,000 lymphocytes were recorded, and data was exported as %parent normalized to %live $CD45^+$ immune cells. Positive populations are denoted by gates on each histogram and * denotes which population each histogram was gated on.

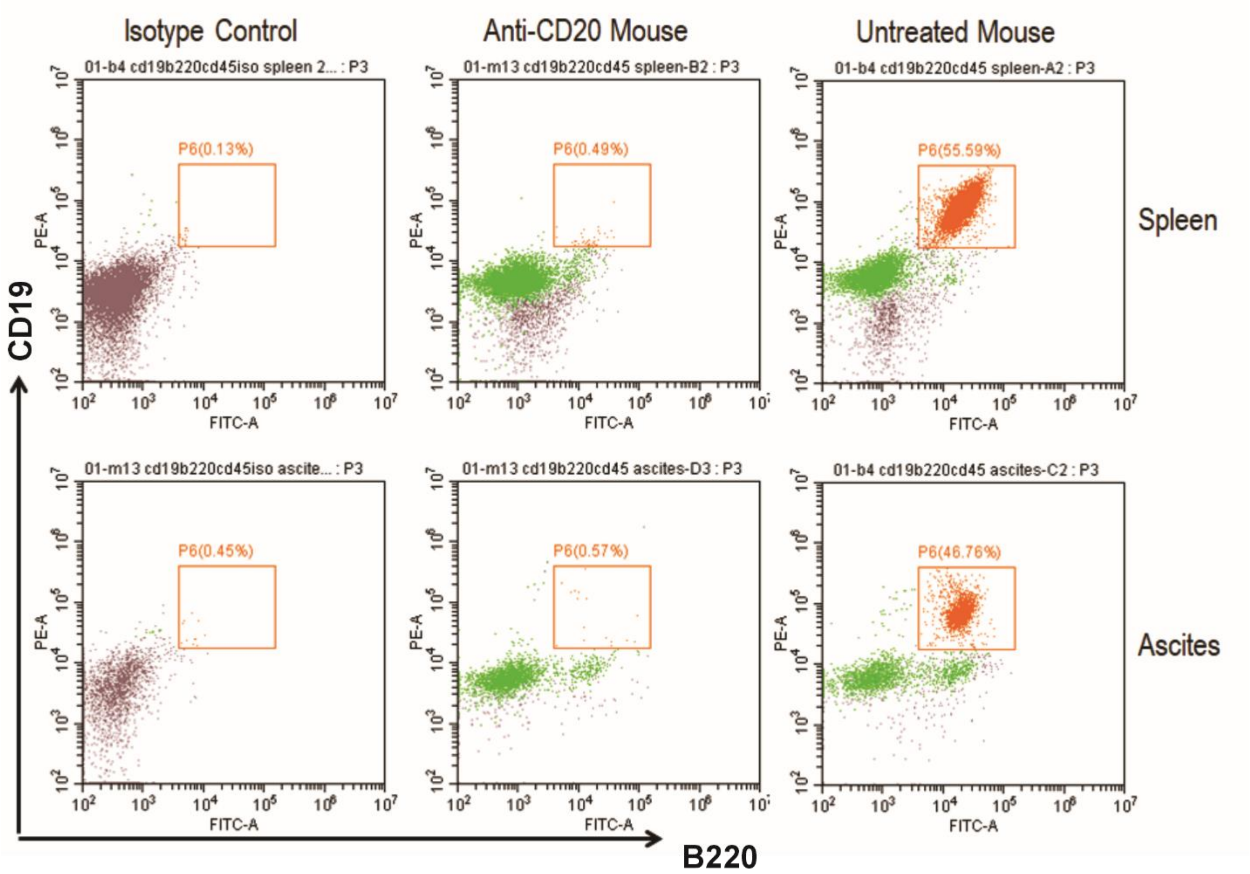


**Fig. S8 Extent of B cell-depletion in spleen and ascites of FVB mice 2 weeks after Anti-CD20 injection.**

Spleens and ascites were harvested from tumor-bearing FVB mice that met protocol-defined endpoints in the extended survival study two weeks after administration of anti-CD20 or control mice without B cell depletion. Splenocytes and ascites cells were stained with fluorophore-conjugated antibodies against CD19 and B220 (along with isotype controls) to compare B cell populations following analysis by flow cytometry. Representative histograms and gating are shown.
